# Co-development of central and peripheral neurons with trunk mesendoderm in human elongating multi-lineage organized gastruloids

**DOI:** 10.1038/s41467-021-23294-7

**Published:** 2021-05-21

**Authors:** Zachary T. Olmsted, Janet L. Paluh

**Affiliations:** State University of New York Polytechnic Institute, College of Nanoscale Science and Engineering, Nanobioscience Constellation, Albany, NY USA

**Keywords:** Pattern formation, Neural patterning, Pluripotent stem cells

## Abstract

Stem cell technologies including self-assembling 3D tissue models provide access to early human neurodevelopment and fundamental insights into neuropathologies. Gastruloid models have not been used to investigate co-developing central and peripheral neuronal systems with trunk mesendoderm which we achieve here in elongating multi-lineage organized (EMLO) gastruloids. We evaluate EMLOs over a forty-day period, applying immunofluorescence of multi-lineage and functional biomarkers, including day 16 single-cell RNA-Seq, and evaluation of ectodermal and non-ectodermal neural crest cells (NCCs). We identify NCCs that differentiate to form peripheral neurons integrated with an upstream spinal cord region after day 8. This follows initial EMLO polarization events that coordinate with endoderm differentiation and primitive gut tube formation during multicellular spatial reorganization. This combined human central-peripheral nervous system model of early organogenesis highlights developmental events of mesendoderm and neuromuscular trunk regions and enables systemic studies of tissue interactions and innervation of neuromuscular, enteric and cardiac relevance.

## Abstract (web summary)

The authors generate EMLOs (elongating multi-lineage organized gastruloids): organoids that self-organize to form compartments with characteristics of the central nervous system, peripheral nervous system, mesenchyme, and gut tube.

## Introduction

Three-dimensional in vitro tissue models called organoids have revealed the astonishing innate capacity of stem cells to self-organize into complex cytoarchitectures resembling in vivo states^1^. Directed single-organoid models of anatomical endpoints of interest, such as regions of the brain, spinal cord (SC), gastrointestinal tract, and heart, are rapidly becoming indispensable to biomedical research for modeling physiology, development, disease, aging, and toxicity^2^. Dual-fated trunk organoids have been applied to model the neuromuscular circuit, demonstrating an ability to recapitulate several key aspects such as interneurons, motor neurons, and the neuromuscular junction (NMJ) with functional output^3^. However, beyond organogenesis, the study of multi-tissue interactions in human models will benefit from an ability to better recapitulate the morphogenetic complexity of embryogenesis. As such, a shift towards new organoid models, deemed gastruloids, which more accurately reflect early mammalian development in a multi-lineage, embryo-like context has emerged^4^. Pioneered primarily using mouse embryonic stem cells (mESCs)^5^, and in one study using human embryonic stem cells (hESCs) for anterior–posterior organization^6^, gastruloids have been applied in vitro to demonstrate symmetry-breaking and axial elongation events^5,7^, somitogenesis with the neural tube^8,9^, and cardiogenesis^10^. Yet, although gastruloids provide a means to interrogate developmental processes with unprecedented detail in vitro, no gastruloid study using either mESCs or human stem cells has previously investigated the co-emergence of central and peripheral nervous system correlates by neuronal markers beyond SOX2 expression in the neural tube^9^. Such a system, as developed herein, would constitute a major advance towards investigating central nervous system–peripheral nervous system (CNS–PNS) co-development with multiple organ precursors such as the primitive gut tube, and ultimately for human neuromuscular studies and end-organ innervation.

Here, we describe a CNS–PNS neurodevelopmental model as elongating multi-lineage organized (EMLO) gastruloids using human iPSCs that achieve increased morphogenetic complexity and multi-system physiological relevance versus existing organoid models. We use ethnically diverse hiPSC (ED-hiPSC) lines reprogrammed from African American, Hispanic-Latino, and Asian donors^11,12^ to demonstrate highly reproducible EMLO generation of integrated embryonic tissue precursors. In particular, we distinguish CNS and PNS correlates by SC neuronal subtypes and neural crest cell (NCC) derivatives, respectively, which form in concert with a self-organizing endoderm-derived primitive gut tube. As highly compartmentalized 3D aggregates formed without embedding, EMLOs consist of a posterior compartment with SC dorsal-intermediate neuronal subtype identity by default, and an elongating anterior compartment arising from endoderm and mesoderm that is then populated by NCCs. The primitive gut tube is a central element along with the SC region and peripheral neurons formed by lineage-differentiating NCCs. The surrounding mesenchyme also creates a permissive microenvironment for neuromuscular and cardiomyocyte differentiation^13^. By small molecule interference with germ-layer specification, we disrupt mesenchyme and primitive gut tube formation and elongation, and the corresponding neuronal patterning events in that compartment. We characterize EMLOs over a 40-day period and validate cell and tissue type biomarkers, including biomarkers of NCC lineage diversification, CNS and PNS neurons, and the gut tube along with additional multicellular diversity. We further validate cell-specific gene expression in day 16 EMLOs using single-cell RNA-Seq (scRNAseq). Finally, we demonstrate the presence of the mu opioid receptor (MOR) in EMLOs and the potential utility of EMLO models in pharmacological studies with agents impacting sensory neuronal activity versus spinal motor neurons that lack the receptor. Our unique EMLO approach with human stem cells is an advanced heterogenous 3D culture platform for NCC analysis, trunk endoderm innervation, and neuromuscular studies.

## Results

### Reproducible generation of EMLO gastruloids from African American, Hispanic-Latino, and Asian hiPSC lines

**Fig. 1:**
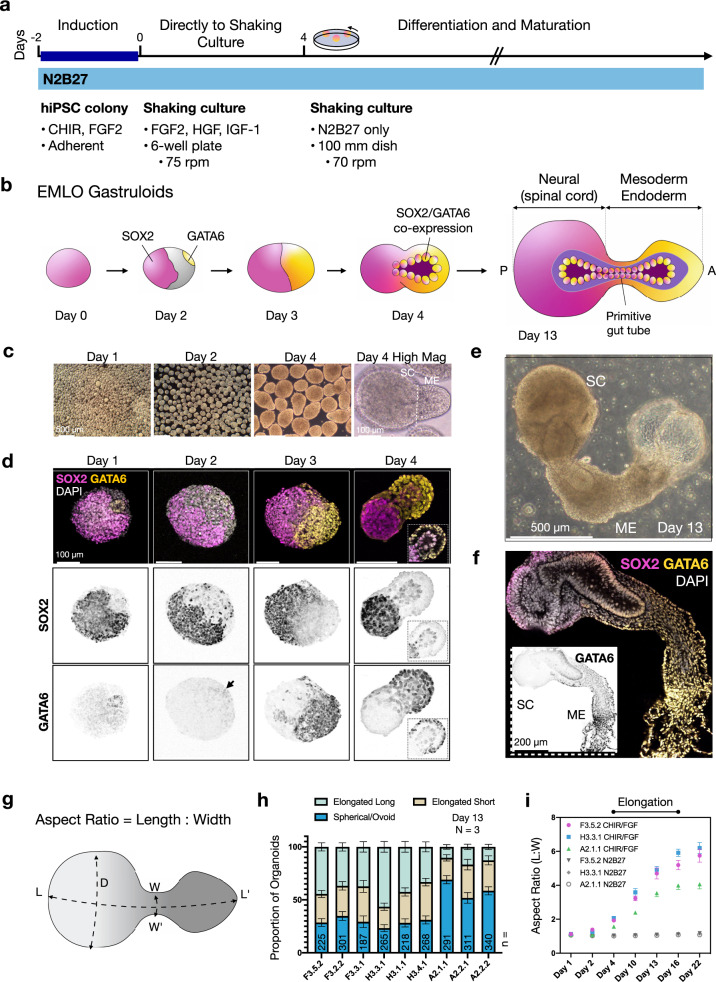
Polarized gene expression and morphology in elongating multi-lineage organized (EMLO) gastruloids. **a** Overview of EMLO gastruloid formation. Intact hiPSC colonies were pretreated with CHIR and FGF2 for 2 days prior to dissociation and transition to shaking culture on day 0. **b** Schematic representation of SOX2 (pink) and GATA6 (yellow) polarized expression in EMLOs over time. Early invagination of SOX2/GATA6 cells precedes the formation of a primitive gut tube-like structure. **c** Phase contrast of early EMLO shaking cultures at days 1, 2, and 4. Early compartmentalization of spinal cord (SC) neural versus mesoderm–endoderm protrusion (ME) can be visualized. **d** SOX2/GATA6 immunofluorescence (IF) of early EMLO aggregates. Day 4 inset depicts SOX2/GATA6 early tube formation (Z-slice). **e** Phase contrast of day 13 EMLO. **f** SOX2/GATA6 IF in day 13 EMLOs with DAPI (gray). SOX2/GATA6 colocalization persists in the primitive gut tube. Inset is GATA6 inverted LUT. H3.3.1 EMLOs were formed in *N* = 11 separate biological repeat experiments over the course of this study with similar results. Phase and IF data were acquired each time **c**–**f**. EMLOs from the other F3.5.2 and A2.1.1 representative lines were separately formed in *N* = 5 biological repeat experiments. All other lines were formed in *N* = 3 repeat experiments. Images are representative of the EMLO populations across repeated experiments. **g** Schematic representation of the EMLO size parameters. Length (L), width (W), and SC diameter (D) were measured. **h** Histogram of the proportion of aggregates that are elongated long (L:W ratio >3.5), elongated short (2 < L:W < 3.5), or spherical/ovoid (L:W < 2) at day 13 using nine ED-hiPSC lines. The plot corresponds to elongation efficiency. EMLO formation using all lines was performed *N* = 3. *n* = number of aggregates measured is shown for each line. **i** Aspect ratio over time in representative ED-hiPSC lines. Elongation after pretreatment (*n* = 153 per line) was compared with elongation in basal medium N2B27 (*n* = 138 per line). Individual scale bars provided. Data reported as (mean ± s.e.m.).

To increase stem cell studies that reflect ethnic diversity (ED) in the United States, we analyzed EMLO gastruloids using previously derived hiPSC lines from our laboratory^11,12,14–16^. Nine ED-hiPSC lines derived from the fibroblasts of three donors of self-designated African American (F3.5.2, F3.2.2, F3.3.1), Hispanic-Latino (H3.3.1, H3.1.1, H3.4.1), and Asian (A2.1.1, A2.2.1, A2.2.2) ethnicities were compared (Supplementary Fig. 1, Supplementary Table 1). EMLOs were generated by modifying a dual-fated neuromuscular organoid (NMO) protocol (Supplementary Fig. 1c–f)^3^ to include mesendoderm fate by altering key initial physicochemical inductive and temporal cues (Fig. 1a; Supplementary Table 2). These modifications allowed retention of primitive mesendoderm^17^ and ectodermal phenotypes during EMLO formation that has not been previously investigated. In brief, intact 2D stem cell colonies were pretreated with CHIR and fibroblast growth factor (FGF2) in N2B27 induction medium for 2 days. After pretreatment, we generated highly uniform 3D starting aggregates (<100 μm diameter, ~300–400 cells per aggregate) by dissociation and immediate transition to low adhesion shaking cultures, bypassing static 96-well plates. FGF2, hepatocyte growth factor (HGF), and insulin-like growth factor-1 (IGF-1) growth factors were included in N2B27 at the time of spontaneous aggregation. Aggregates were split 1:2 after 24 h and maintained to day 4, at which point aggregates were expanded to 100 mm dishes and maintained in N2B27 alone to at least day 22. We monitored morphogenetic properties over time by phase-contrast microscopy and whole-mount immunofluorescence (IF) of tissue-cleared samples (Fig. 1b–i).

SOX2 and GATA6 are two essential transcription factors involved in myriad early developmental polarization events, wherein the individual deletion of each gene is embryonic lethal^18,19^. In EMLOs, we observed polarized gene expression of SOX2 versus GATA6 as soon as 24 h after aggregation (Fig. 1d). A prominent GATA6 domain emerged by day 3, opposite the SOX2 domain as previously observed in human gastruloids^6^, with some interpenetration. Notably, by day 4, we observed asymmetric bipolar aggregates that exhibited single bilaminar protrusions extending out of the SOX2 domain. Although the outer cell layer was predominately GATA6+, the inner invaginated layer co-expressed GATA6 and SOX2. This mixed factor protrusion continued to elongate between days 4 and 16 (Fig. 1e, day 13), a critical EMLO elongation period, and ultimately produced a laminated tube-like structure that retained SOX2/GATA6 double-positivity (Fig. 1f). In general, although there is overlap during initial EMLO polarization events (Supplementary Fig. 2e), the SOX2 domain matures to have primarily neural SC identity that is posterior, and the GATA6 domain matures to have primarily mesoderm and endoderm identity (mesoderm–endoderm, ME), and is anterior. We use the terminology “domain” when referring to early biomarkers, and “compartment” when referring to the tissue structure as a whole. To exclude any contribution of aggregate fusion to EMLO gross morphological complexity, we tracked individual aggregates and monitored elongation (Supplementary Fig. 1g). By this approach, we also observed the emergence of a dilated vesicular structure in the anterior EMLO pole of ME that extended in length and in size to day 13 (Fig. 1e), after which this region resembled amorphous sacs lined by GATA6+ cells (Supplementary Fig. 1h).

To evaluate reproducibility of EMLOs, we measured gross size parameters and assessed formation efficiency using the morphologically distinct hourglass shape in all nine ED-hiPSC lines (Fig. 1g–i). The major axis length (L) was measured between the most distant points of the posterior and anterior poles, while the minor axis after elongation was defined as the width (W) of the ME compartment near the SC exit point (Fig. 1g). We categorized day 13 EMLOs as elongated long, elongated short, or spherical/ovoid based on aspect ratio in *N* = 3 repeated formation experiments (Fig. 1h). Replicate ED-hiPSC lines had more similar elongation efficiencies, with greater differences seen between cohorts. Among all lines, H3.3.1 had the highest efficiency of elongation (52.8% elongated long, 20.8% elongated short, 73.6% total). Given similar EMLO formation efficiencies within replicate lines, we chose one line from each cohort to investigate further that were F3.5.2, H3.3.1, and A2.1.1. Representative lines were previously extensively characterized genomically, transcriptomically, functionally, and by protein biomarker in multi-lineage differentiation platforms^11,12^. All three lines form teratomas in nude mice^11^. Aspect ratios in EMLOs were measured over time and compared to aggregates formed without CHIR/FGF2 pretreatment (N2B27 pretreatment only) (Fig. 1i). Elongation was not observed in cerebral organoids generated from anterior NSCs, or in suspension neurospheres that used caudal scNSCs as starting material (Supplementary Fig. 3). As well, EMLO elongation was prevented by addition of 1 μM retinoic acid starting on day 2, presumably owing to premature conversion to neuroectoderm (Supplementary Fig. 3a, c). Culture conditions applied to promote or inhibit EMLO formation and elongation are summarized (Supplementary Table 2).

### Self-organization of a primitive gut tube-like structure follows initial polarization in EMLOs

Although a 3-day pretreatment of hiPSCs seeded as single cells yields homogenous adherent cultures of SOX2/Bra neuromesodermal progenitors (NMPs) that benefit NMO formation (Supplementary Fig. 1c–f)^3^, our shorter induction period yielded a less-restricted mesendoderm-like cellular starting material characterized by expression of SOX2, Bra, and the definitive endoderm marker FOXA2/HNF-3β (Supplementary Fig. 2a–d). By IF of day 3 early EMLOs (Supplementary Fig. 2e), we observed compartmentalized expression of transcription factors that orchestrate multi-lineage gene regulatory networks in development. Similarly to gastruloids^6,7^, EMLO morphological asymmetry and elongation were preceded by asymmetric polarization of cellular pools within aggregates. Cells expressing definitive endoderm markers SOX17 and FOXA2 were detected within the SOX2 domain as previously observed^5,6^. Bra+ cells that precede mesoderm were localized to the tip of the posterior pole by day 3. Expression of CDX2, master upstream regulator of caudal *Hox* genes, occurred opposite the GATA6 domain^6^. Together, these data suggest that multiple germ layers co-emerge in early EMLOs and contribute to downstream morphology and reproducibility. Early cytological complexity was evident and nearly indistinguishable in all three representative lines.

**Fig. 2:**
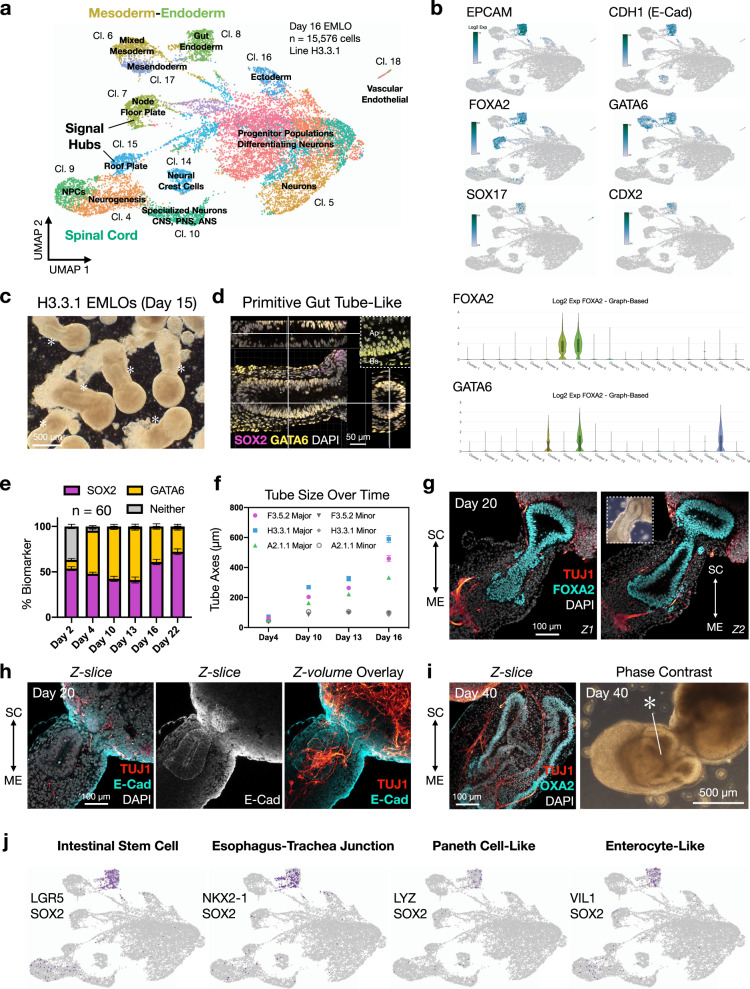
Reproducible self-organization of a primitive gut tube-like structure. **a** Single-cell RNA-Seq (scRNAseq) cluster annotation of H3.3.1 day 16 EMLOs (*n* = 15,576 cells, *N* = 1 biological sample) plotted with UMAP. **b** Canonical gut tube epithelial genes by scRNAseq with violin plots for *FOXA2* (1,707/15,576 cells) and *GATA6* (1,244/15,576 cells) (log2Exp). Violin plot statistics are as follows: *FOXA2* (cluster 7 max = 5.36, min = 0, median = 1.59, q1 = 1, q3 = 2.32; cluster 8 max = 5.25, min = 0, median = 2, q1 = 1, q3 = 2.59), *GATA6* (cluster 6 max = 3.32, min = 0, median = 0.66, q3 = 1; cluster 8 max = 3.91, min = 0, median = 1.06, q3 = 1.58; cluster 17 max = 4.09, min = 0, median = 1, q3 = 1.58). **c** Phase contrast of EMLO shaking cultures at day 15. Asterisk indicates gut tube (*). **d** Multi-dimensional visualization of SOX2 (magenta) and GATA6 (yellow) primitive gut tube in day 13 EMLO. Sagittal and transverse planes are shown. High magnification Z-slice depicts mitotic figures in apical (Ap) but not basal (Bs) epithelium. **e** SOX2 and GATA6 distribution as percent biomarker calculated from maximally projected Z-stacks of whole EMLOs over time. *n* = 60 total EMLOs measured (H3.3.1). **f** Length of gut tube major and minor axes over the elongation period in the three representative lines (*n* = 47 EMLOs measured per line). **g** Z-slices depict robust FOXA2 (cyan) expression in gut tube and associated TUJ1 (red) neuronal fibers at day 20. Inset is phase contrast. **h** Z-slice of CDH1/E-cadherin (cyan) and TUJ1 (red) IF at day 20. Maximally projected TUJ1 Z-volume is overlaid onto E-cad Z-slice to show 3D spatial relationship (right). **i** Increased gut tube morphological complexity at day 40 by FOXA2 and TUJ1 IF and phase contrast. Asterisk highlights meandering gut tube at this time point (*). **j** Identification of specified gastrointestinal cell types in day 16 EMLOs by scRNAseq that are intestinal stem cell (LGR5/SOX), esophagus–trachea junction (NKX2-1/SOX2)^13^, Paneth cell-like (LYZ/SOX2), and enterocyte-like (VIL1/SOX2). Individual scale bars provided. H3.3.1 EMLOs were formed in *N* = 11 separate biological repeat experiments over the course of this study with similar results. Phase and IF data were acquired each time (**c**, **d**, **g**–**i**). Data reported as (mean ± s.e.m.).

We performed scRNAseq analysis on EMLOs at day 16 in formation using the H3.3.1 line (*n* = 15,576 cells) (Fig. 2a) and applied 10× Genomics Loupe analysis software. Eighteen clusters were produced and were annotated based on canonical gene biomarkers. Gene lists used for annotation are provided as a separate data file. At this time point, several important clusters were identified including gut endoderm (cluster 8), mixed mesoderm (cluster 6), floor plate-like and roof plate-like signaling hubs (clusters 7 and 15, respectively), neural tube and SC-like regions (clusters 4, 5, 9, 10), NCCs (cluster 14), ectoderm (cluster 16), differentiating neural progenitor populations (clusters 1–3, 11, 12), and a small vascular/endothelial cluster (cluster 18). A robust, self-organizing tube-like structure spanned the SC and ME compartments in ~100% of EMLOs that underwent elongation. This structure did not occur in the non-elongated spherical/ovoid subset. To determine the identity of this tube structure, we investigated individual transcripts within clusters and performed additional imaging analysis (Fig. 2b–j). This architectural feature was visible by phase-contrast microscopy (Fig. 2c). By Z-stack 3D reconstruction (Fig. 2d), we show that the GATA6 expressing tube is indeed a continuous, cylinder-like structure with a central lumen containing mitotic figures at the apical aspect (see also Supplementary Fig. 4, Supplementary Movie 1).

We determined that the structure has gut tube epithelial identity by coupling canonical gene expression in cluster 8 for definitive endoderm (*EPCAM*, *CDH1*/E-cadherin, *FOXA2*, *GATA6*, *SOX17*, *CDX2*) (Fig. 2b) with IF of FOXA2 (Fig. 2g) and E-Cadherin (Fig. 2h). E-Cadherin was expressed in the tube in both SC and ME compartments indicating definitive endoderm (Fig. 2h). GATA6 was strictly excluded from SC, though highly expressed both in the palisading tube epithelium and the surrounding, loose mesenchyme-like tissue. These two regions in ME were separated by acellular space indicating basement membrane. Similarly, *FOXA2* and *GATA6* were found to colocalize in cluster 8. Based on these data, we classified this structure as a putative primitive gut tube, hereon simply referred to as the gut tube. Gut tube identity was supported by low level SOX2 co-expression with high GATA6^5,20,21^. To determine the approximate relative proportion of neural versus non-neural lineages, we quantified the areas of SOX2 versus GATA6 domains in EMLOs over time in maximally projected images from complete Z-stacks (Fig. 2e). SOX2 basal expression in the gut tube was excluded for the purpose of these measurements. We also measured the gut tube major and minor axes over time (Fig. 2f), which correlated with total EMLO elongation and aspect ratio (Fig. 1i) in support of a critical elongation period from day 4 to 16. A FOXA2+ gut tube was evident by day 8 (Supplementary Fig. 2f), though became increasingly morphologically complex in close spatial relation to TUJ1 neuronal fibers (Fig. 2g–i, Supplementary Movie 2) and late-stage day 40 EMLOs had meandering gut tube morphologies (Fig. 2i). Using Boolean filter rules of day 16 scRNAseq data, we identified distinct gastrointestinal cell populations in cluster 8 that are intestinal stem cell (*LGR5*/*SOX2*), esophagus–trachea junction (*NKX2-1*/*SOX2*)^13^, Paneth cell (*LYZ*/*SOX2*), and enterocyte (*VIL1*/*SOX2*). A qualitative summary of EMLO biomarker distribution analyzed by IF is provided (Supplementary Table 3).

### EMLO gastruloids have default dorsal-intermediate SC identity that can be ventralized

**Fig. 3:**
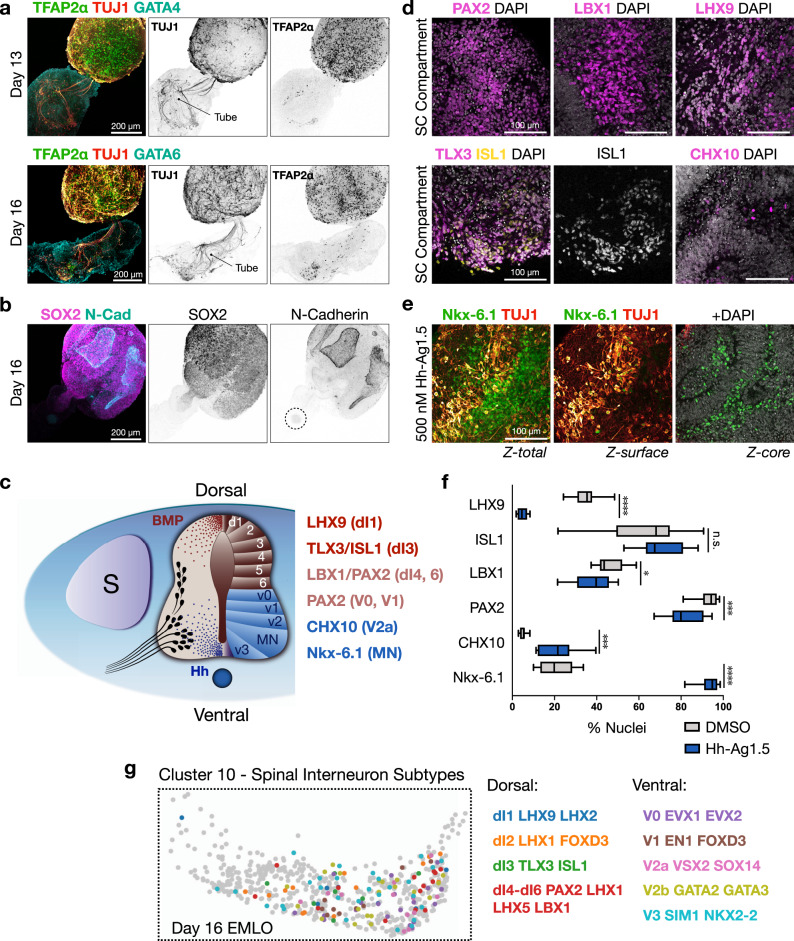
Default dorsal-intermediate spinal cord identity in EMLOs can be ventralized. **a** IF of day 13 and day 16 H3.3.1 EMLOs depicts TFAP2α (green) and GATA4 (cyan) (top), and TFAP2α (green) and GATA6 (cyan) (bottom), counterstained with TUJ1 (red). Position of gut tube is shown to highlight TFAP2α cell migration. **b** N-cadherin is restricted to SOX2 domain and gut tube anterior pole (dotted circle). **c** Schematic representation of spinal cord domains along dorsal–ventral axis with ventral (Hh) and dorsal (BMP) morphogen signaling gradients. Somite (S) and notochord (ventral, blue circle) are shown. **d** Day 22 spinal cord interneuron domain subtypes PAX2 (dI4, dI6, V0, V1), LBX1 (dI4-dI6), LHX9 (dI1), TLX3/ISL1 (dI3), CHX10 (V2a) in neural SC compartment. **e** Nkx-6.1 expression at day 22 suggests ventralization of spinal cord phenotype by addition of 500 nM Hh-Ag1.5 on day 10. **f** Quantification of neuronal subtype biomarkers in H3.3.1 EMLOs under control (DMSO, gray) or ventralizing (Hh-Ag1.5, blue) conditions. LHX9 (****p < 0.0001, *t* = 11.66, *df* = 15), ISL1 (n.s. *p* = 0.36, *t* = 0.9480, *df* = 19), LBX1 (*p = 0.047, *t* = 2.133, *df* = 18), PAX2 (***p = 0.0002, *t* = 4.352, *df* = 23), CHX10 (***p = 0.0005, *t* = 4.518, *df* = 14), Nkx-6.1 (****p < 0.0001, *t* = 23.09, *df* = 17) by unpaired two-tailed *t* test. *n* = 6000 cells minimum counted per condition from *N* = 1 experiment (Source Data file). The box and whisker plot statistics are as follows: LHX9 (Hh-Ag1.5: max = 8.35, min = 1.79, median = 4.52, q1 = 2.51, q3 = 6.97; DMSO: max = 48.50, min = 24.25, median = 35.45, q1 = 37.80; q3 = 30.86); ISL1 (Hh-Ag1.5: max = 88.07, min = 52.89, median = 67.52, q1 = 63.34, q3 = 80.90; DMSO: max = 90.68, min = 21.66, median = 68.15, q1 = 49.29, q3 = 74.60); LBX1 (Hh-Ag1.5: max = 98.24, min = 81.02, median = 94.29, q1 = 90.48, q3 = 96.91; DMSO: max = 50.24, min = 21.43, median = 39.72, q1 = 30.74, q3 = 45.77); PAX2: (Hh-Ag1.5: max = 94.68, min = 67.22, median = 79.94, q1 = 75.94, q3 = 90.63); CHX10 (Hh-Ag1.5: max = 39.62, min = 10.03, median = 19.87, q1 = 13.29, q3 = 26.98; DMSO: max = 8.30, min = 2.87, median = 5.54, q1 = 3.67, q3 = 5.75); Nkx-6.1 (Hh-Ag1.5: max = 98.57, min = 81.78, median = 94.81, q1 = 90.99, q3 = 97.16; DMSO: max = 33.67, min = 10.03, median = 19.87, q1 = 13.29, q3 = 28.34). **g** Dorsal (dI) and ventral (V) interneuron subtypes in scRNAseq neuronal cluster 10. Color-coded biomarker combinations are listed. Histogram data reported as (mean ±s.e.m.). Individual scale bars provided. For scRNAseq, *N* = 1 biological sample was analyzed with *n* = 15,576 cells. For IF, CDH2, GATA4, GATA6, TUJ1, and TFAP2α immunostain was performed for each of the *N* = 11 H3.3.1 EMLO formation experiments with similar results **a**, **b**. The experiment in **d**–**f** was performed *N* = 1 time with a parallel analysis performed using the scRNAseq data.

We corroborated EMLO trunk identity by scRNAseq data, identifying an SC-like region in clusters 4, 9, 10, floor plate- and roof plate-like signaling hubs, and a suite of *Hox* genes that are known to be absent in anterior neuroectoderm. EMLOs exhibited a distinct lack of anterior neuroectodermal transcripts *OTX2*, *FOXG1*, and *TBR2* (Supplementary Fig. 5) and these proteins were not observed by IF. To evaluate cell and lineage identity in the EMLO neural SC compartment, we performed IF of neurons and neural crest cells, or NCCs (Fig. 3). By IF of β-III-tubulin (TUJ1), a neuron-specific β-tubulin isotype, we observed a dense population of neurons in SC that colocalized with TFAP2α, indicating NCC lineages and/or developing GABAergic interneuron progenitors. A small subset of neurons projected into the ME compartment, shown within GATA4+ tissue at day 13 (Fig. 3a top). Notably, GATA4 was excluded from the gut tube but highly expressed in the surrounding mesenchyme. At day 16, we demonstrated that the projecting neurons enveloped the GATA6+ gut tube (Fig. 3a bottom). A small number of TFAP2α nuclei were observed as well in close association with neuronal fibers in ME, indicating a migratory population of NCC-derived neurons or fate-biased non-neuronal NCCs that migrate along or in proximity to neuronal fibers. The number of TFAP2α nuclei in ME increased over time, whereas TUJ1 signal increased in both SC and ME. By day 16, TFAP2α+ neurons self-organized into a single ganglionic structure at the base of the gut tube. The neuroanatomical term ganglion is used in the literature to describe neural structures in organisms that range from invertebrate to mammals, and is used here similarly to mean a peripheral structure that contains a collection of neuronal cell bodies.

To validate the neural identity of SC, we showed that neural cadherin (N-cadherin, or CDH2) is restricted to SC as opposed to the gut tube and primarily in large neural rosettes (Fig. 3b). Important, however, is that N-cadherin signal was detected towards the base of the gut tube, suggesting a distal region of neural character in support of a peripheral ganglionic structure. We analyzed a suite of CNS markers to further characterize the SC compartment. We detected abundant expression of several transcription factors used in vivo to delineate developing SC progenitor and interneuron subtype domains along the dorsal–ventral axis (Fig. 3c, f)^22^. These were LHX9 (dI1), TLX3/ISL1 (dI3), LBX1 (dI4-dI6), PAX2 (dI4, dI6, V0, V1), CHX10 (V2a), and Nkx-6.1 (MN). By counting nuclei in H3.3.1 EMLOs, we classified the day 22 SC as having primarily dorsal-intermediate SC regional identity by default owing to high levels of LHX9, PAX2, and LBX1 in the context of lower CHX10 and Nkx-6.1 (Fig. 3d, f). PAX2 is an important marker of the intermediate SC as it spans the dorsal to ventral domains. ISL1 is expressed in both dorsal dI3 interneurons and ventral spinal motor neurons, and so we used the additional marker TLX3 indicating dI3. Given the susceptibility of SC regional identity to changes in opposing BMP (dorsal) and Hh (ventral) morphogenetic signaling gradients^23^, we hypothesized that addition of the potent Hh small molecule agonist Hh-Ag1.5 may shift the relative proportion of interneuron subpopulations by ventralization. Accordingly, we supplemented N2B27 with 500 nM Hh-Ag1.5 starting at day 10 and observed a significant increase in the percentage of CHX10 (***p = 0.0005) and Nkx-6.1 (****p < 0.0001) positive nuclei in day 22 EMLOs versus dimethyl sulfoxide (DMSO) control (Fig. 3e, f), as well as significant decreases in PAX2 (***p = 0.0002), LBX1 (*p = 0.047), and LHX9 (****p < 0.0001). As additional evidence, we performed a similar analysis using biomarker combinations for the dorsal and ventral SC interneuron domains using the scRNAseq data set (Fig. 3g). Cluster 10 contained the greatest proportion of these neuron types and was expanded for visualization. Together, these data suggest that EMLOs have default dorsal-intermediate SC identity, and that populations of neuronal progenitors and subtypes can be further manipulated by exogenous signaling interventions.

### EMLOs specialize to generate motor, sensory and autonomic neurons, and neurotransmission phenotypes

**Fig. 4:**
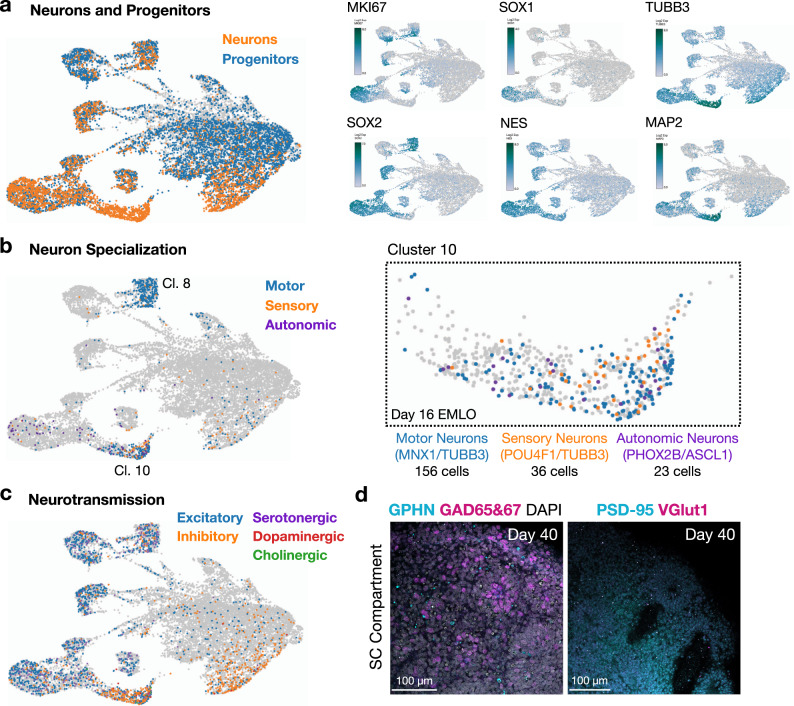
Quantification of neuron specialization and neurotransmission phenotypes in EMLOs. **a** Distribution of neural progenitors (*TUBB3* OR *MAP2*, blue) and neurons (*TUBB3* AND *MAP2*, orange) in day 16 H3.3.1 EMLOs by scRNAseq (left). Canonical neuronal differentiation genes are shown (log2Exp) (right). **b** Proportion of specialized neurons generally classified as motor (*MNX1*/*TUBB3*, blue), sensory (*POU4F1*/*TUBB3*, orange), and autonomic (*PHOX2B*/*ASCL1*, purple) at day 16. Cluster 10 is expanded. **c** Proportion of neurotransmission phenotypes generally classified as excitatory (blue), inhibitory (orange), serotonergic (purple), dopaminergic (blue), and cholinergic (green) at day 16. **d** Inhibitory (GPHN in cyan, GAD65&67 in magenta) and excitatory (PSD-95 in cyan, VGlut1 in magenta) neurotransmission biomarkers in SC at day 40 (*N* = 2 repeat experiments). Individual scale bars provided.

We further applied scRNAseq and biomarker imaging to distinguish neural progenitors, types of neurons, and modes of neurotransmission (Fig. 4). We distinguished neural progenitors (*TUBB3* OR *MAP2*; 6700 cells) from committed neurons (*TUBB3* AND *MAP2*; 3030 cells). By this filter, cluster 10 contained the highest proportion of neurons, supported by individual analysis of genes involved in neuronal differentiation (Fig. 4a). To determine the transcriptional phenotypes of neurons in this cluster, we applied further generalized filters with genes for motor neurons (*MNX1* AND *TUBB3*; 156 cells), sensory neurons (*POU4F1* AND *TUBB3*; 36 cells), and autonomic neurons (*PHOX2B* AND *ASCL1*; 23 cells) (Fig. 4b). Cluster 10 was expanded for easy visualization. Interestingly, by the *MNX1*/*TUBB3* filter, the motor neuron transcriptional phenotype was also abundant in cluster 8. Using known genes for neurotransmission, we identified modes of neurotransmission in the day 16 EMLOs that are broadly excitatory, inhibitory, serotonergic, dopaminergic, and cholinergic (Fig. 4c). At day 40, proteins for inhibitory (GPHN/GAD65&67) and excitatory (PSD-95/VGlut1) pre- and post-synaptic machineries were detected as colocalized puncta in SC (Fig. 4d). These data suggest that neuronal phenotypes begin to emerge by differentiation in EMLOs, even at the day 16 time point that is early by standard neuronal differentiation protocols, and assemble pre- and post-synaptic machinery as they mature.

### Neuronal cytoarchitectures are established in close relationship to the gut tube

**Fig. 5:**
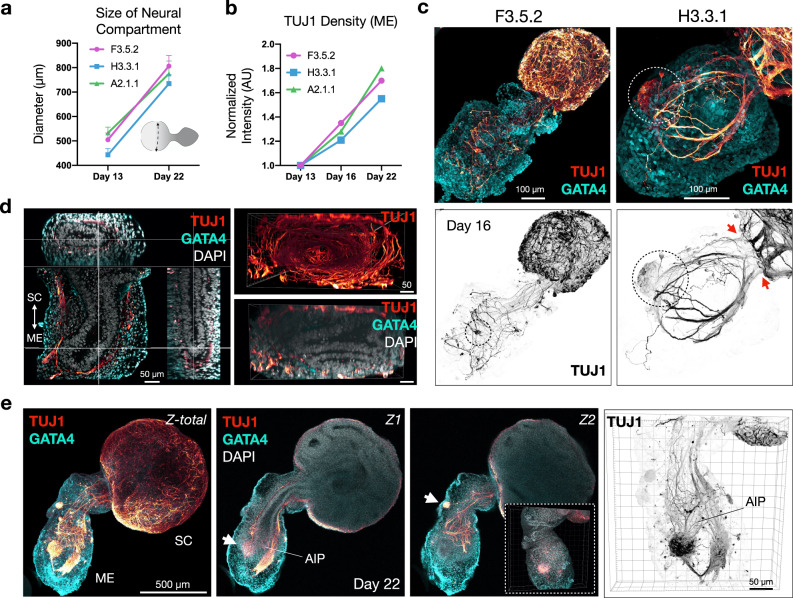
Neuronal cytoarchitectures are reproducibly patterned in close relationship to the gut tube. **a** Change in diameter of spinal cord (SC) compartment from days 13 to 22 (*n* = 47 EMLOs measured per line). **b** Change in TUJ1 density by normalized fluorescence of Z-stack volumes between days 13, 16, and 22 (*n* = 3 EMLOs per time point per line). **c** TUJ1 (red) and GATA4 (cyan) IF of F3.5.2 and H3.3.1 day 16 EMLOs. Peripheral ganglion formation (dotted circle) and neuronal bottleneck (red arrows) are appreciated. ImageJ inverted LUT (TUJ1) was used for black and white images. **d** Multi-dimensional visualization of gut tube-neuron interaction in H3.3.1 day 16 EMLO. Sagittal and transverse planes of gut tube subsumed by neural processes (left); end-on view of neuronal tunnel (TUJ1+, top) about gut tube (DAPI, bottom). **e** TUJ1/GATA4 of H3.3.1 day 22 EMLO. Maximally projected Z-stack (left, Z-total) is shown with two Z-slices (Z1, Z2). SC and ME labeled for orientation. Peripheral ganglia form in close proximity to gut tube anterior intestinal portal-like (AIP) region. Z2 inset depicts 3D undulating GATA4 exterior with interpenetrating neurons. High magnification TUJ1 reconstruction shown (right). For IF, GATA4 and TUJ1 staining was performed in each of the *N* = 11 H3.3.1 EMLO formation experiments with similar results **c**–**e**. Individual scale bars provided. Data reported as (mean ±s.e.m.).

Given the close physical relationship between neurons and the gut tube in EMLOs, we sought to further elaborate and quantify this interaction. In parallel with an increase in the diameter of the SC compartment over time (Fig. 5a), we measured a corresponding increase in TUJ1 density in ME from days 13 to 22 (Fig. 5b). Normalized TUJ1 fluorescence was computed from complete Z-stack volumes of this isolated region as a proxy for TUJ1 density. At day 16, the early formation of peripheral ganglia was observed in addition to a neuronal bottleneck effect at the transition zone between SC and ME (Fig. 5c), and using 3D reconstruction of high magnification Z-stacks, it is clear that neuronal fibers in ME completely envelop the gut tube, but are largely excluded from the lumen and the basal lamina (Fig. 5d; Supplementary Movie 3). In a subset of EMLOs, scant TUJ1 fibers were detected within the lumen. By comparison of maximally projected Z-stacks to individual Z-slices in EMLOs at day 22, we demonstrated the retained envelopment of the gut tube and the primary large peripheral ganglion, in addition to the establishment of smaller satellite ganglia distributed throughout the ME in support of advancing neural pattern complexity (Fig. 5e). By 3D reconstruction of the EMLO exterior, an undulating GATA4 + ME surface interpenetrated by neuronal fibers and ganglia was evident. We refer to the anterior pole of the gut tube as the anterior intestinal portal-like region, or AIP.

### Gut tube and mesoderm contribute to EMLO elongation and neuronal pattern formation

**Fig. 6:**
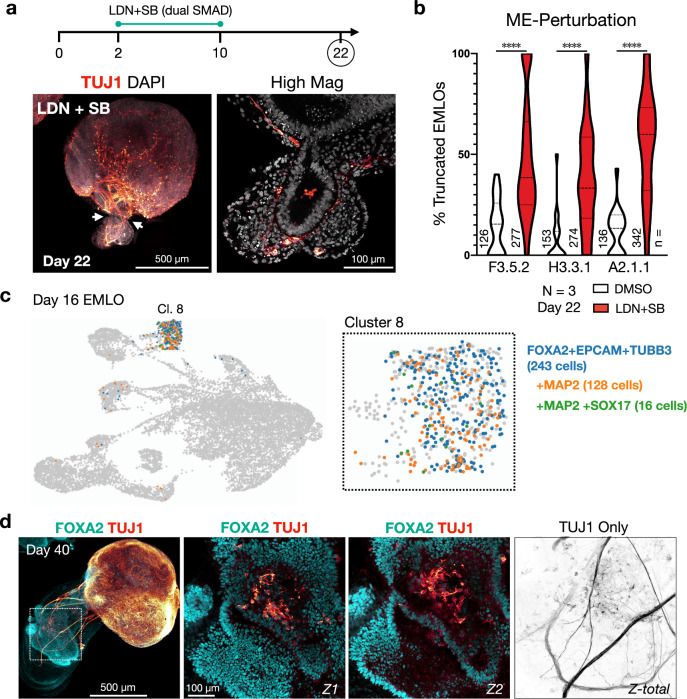
Gut tube formation events impact neuronal patterning. **a**–**b** Dual SMAD small molecule inhibitors LDN 193189 (LDN) and SB 431542 (SB) added before the EMLO elongation phase opposes mesenchymal and endodermal elongation **a**, and was quantified in **b** in representative ED-iPSC lines F3.5.2 (****p < 0.0001, *t* = 4.518, *df* = 61), H3.3.1 (****p < 0.0001, *t* = 7.293, *df* = 61), A2.1.1 (****p < 0.0001, *t* = 6.723, *df* = 61) by unpaired two-tailed *t* test. *n* = number of EMLOs counted (day 22). *N* = 3 biological replicates performed with similar results. Eight to 45 fields were analyzed per condition and data were quantified per field. Violin plot statistics are as follows: F3.5.2 (DMSO: max = 40, min = 0, median = 15.48, q1 = 0, q3 = 25.89; LDN + SB: max = 100, min = 0, median = 15.48, q1 = 25, q3 = 66.25); H3.3.1 (DMSO: max = 50, min = 0, median = 0, q1 = 0, q3 = 11.90; LDN + SB: max = 100, min = 0, median = 33.33, q1 = 18.33, q3 = 58.57); A.2.1.1 (DMSO: max = 48.86, min = 0, median = 13.39, q1 = 0, q3 = 20; LDN + SB: max = 100, min = 0, median = 60, q1 = 32.05, q3 = 73.21). **c** Identification of competing neuronal versus endodermal transcriptional programs in cluster 8 in day 16 H3.3.1 EMLOs by scRNAseq. Canonical endoderm markers *FOXA2* and *EPCAM* were co-expressed with *TUBB3* (243 cells). A subset of *FOXA2*/*EPCAM*/*TUBB3* triple-positive cells (blue) also expressed *MAP2* (128 cells, orange) and then further expressed *SOX17* (16 cells, green). Cluster 8 is expanded. This phenomenon was previously described in vivo for enteric nervous system development^24^. **d** IF of FOXA2 and TUJ1 in day 40 H3.3.1 EMLOs. High magnification Z-slices depict the emergence of FOXA2 + neurons in support of the dual embryonic origin model of the mammalian ENS. FOXA2 and TUJ1 staining on day 40 H3.3.1 EMLOs was performed in *N* = 3 biological replicate experiments with similar results. Individual scale bars provided. Data reported as (mean ± s.e.m.).

To determine whether mesoderm and endoderm have a role in EMLO elongation, we performed a pharmacological inhibition experiment (Fig. 6a, b). We introduced dual SMAD inhibition by small molecules LDN 193189 (200 nM) and SB 431542 (10 μM) to inhibit mesoderm and endoderm progression during the elongation period. LDN and SB were added at day 2 in EMLO formation, a time point near the establishment of sub-aggregate polarized domains, and maintained until day 10. EMLO morphology was quantified at day 22 versus DMSO controls. At this time point, a significant proportion of EMLOs exhibited truncated ME compartments in all three lines (Fig. 6b). *N* = 3 repeat experiments were performed. Notably, SC cytoarchitecture that is characterized by the emergence of neurons at the base of rosettes and a bottleneck effect in the transition zone was not affected by this intervention. However, we did not detect peripheral ganglionic structures in truncated aggregates. These results suggest that EMLO elongation may occur due to ME-driven processes as opposed to NMP-driven processes of axial elongation. Although EMLOs were formed from cells co-expressing SOX2, Bra, and FOXA2 (Supplementary Fig. 2c, d), NMPs were not detected at the day 16 time point by scRNAseq, suggesting that NMPs are transitory and may precede this stage. These multiple features distinguish EMLOs from all other previously described gastruloid models^5,6^.

To follow-up on motor neuron transcriptional programs observed in the scRNAseq cluster 8 (gut tube epithelium), we performed additional sub-cluster analysis (Fig. 6c). Interestingly, a subset of cells expressing epithelial transcripts *FOXA2* and *EPCAM* also expressed neuronal *TUBB3* (243 cells). A further subset of these *FOXA2*/EPCAM/TUBB3 cells expressed neuronal *MAP2* (128 cells), and then the additional endodermal marker *SOX17* (16 cells). We performed *FOXA2* and TUJ1/*TUBB3* IF on day 40 EMLOs and identified a subset of FOXA2 cells in the epithelium that also strongly expressed TUJ1 with neuronal morphology (Fig. 6d). It was recently shown in vivo in mice that enteric neurons have dual embryonic origins from both neural crest and endoderm^24^. Our result may represent a similar phenomenon, and if so, EMLOs would constitute the only human model system currently available to further interrogate this developmental process.

### Neural crest cells position to form neuronal networks along the gut tube

**Fig. 7:**
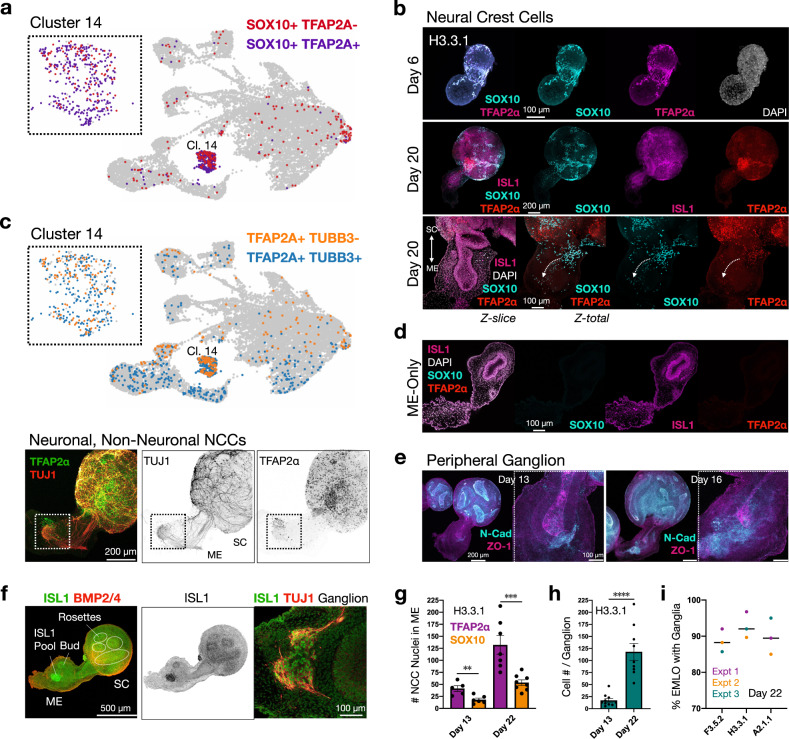
Diverging NCC lineages populate the ME. **a** Two *SOX10-*expressing neural crest cell (NCC) populations are distinguished by *TFAP2A* expression (purple) and non-expression (red) in day 16 EMLOs by scRNAseq. NCC cluster 14 is expanded. **b** TFAP2α (magenta) and SOX10 (cyan) double-positive cells in day 6 H3.3.1 EMLO (top) and diverging NCC lineages by distinct SOX10/TFAP2α immunostaining in day 20 EMLO (middle). High magnification representative day 20 EMLO is shown (bottom). White dotted arrows depict potential NCC migration about the gut tube. **c** Two *TFAP2A-*expressing NCC populations are distinguished by *TUBB3* expression (blue) and non-expression (orange) (top). The NCC cluster 14 is expanded. The subsets of putative neuronal (TFAP2α+ in green, TUJ1+ in red) and non-neuronal (TFAP2α+, TUJ1−) NCCs are visualized as well by IF that is best seen at the ganglion in the white dotted box (bottom). TFAP2α signal is higher outside of the ganglion. **d** EMLOs lacking the spinal cord (SC) compartment does not contain NCCs, supporting an origin in SC. **e** N-cadherin (cyan) expression restricted to SC and the gut tube anterior pole, counterstained with ZO-1 (magenta) and shown at days 13 and 16. High magnification images provided. **f** ISL1 (green) and BMP2/4 (ref) IF in day 22 H3.3.1 EMLOs. ISL1+ cellular pools in proximity to the gut tube are observed in both lines, and increased expression in budding regions of the tube versus non-budding regions. High magnification ISL1 colocalization with TUJ1 is shown (right). NCC biomarkers were stained in H3.3.1 EMLOs in *N* = 8 of 11 formation experiments, and *N* = 3 formation experiments for the other representative lines. **g** Histogram of TFAP2α (purple) and SOX10 (yellow) positive nuclei in mesoderm–endoderm (ME) over time at day 13 (*n* = 5 EMLOs TFAP2α, *n* = 6 EMLOs SOX10) versus day 22 (*n* = 7 EMLOs TFAP2α, *n* = 8 EMLOs SOX10) in H3.3.1 EMLOs. (unpaired two-tailed *t* test **p = 0.0033, *t* = 3.814, *df* = 10 TFAP2α; ***p = 0.0004, *t* = 4.873, *df* = 12). **h** Plot of cell number (DAPI/TUJ1) per ganglion over time at day 13 (*n* = 10 EMLOs) versus day 22 (*n* = 9 EMLOs) in H3.3.1 EMLOs (****p < 0.0001, *t* = 5.854, *df* = 17). Data reported as (mean ±s.e.m.). **i** Histogram quantification of EMLOs with neuronal ganglionic structures in ME in F3.5.2 (*n* = 25), H3.3.1 (*n* = 31), and A2.1.1 (*n* = 19) ED-hiPSC lines at day 22 (median). Colors denote *N* = 3 separate formation experiments. Individual scale bars provided.

**Fig. 8:**
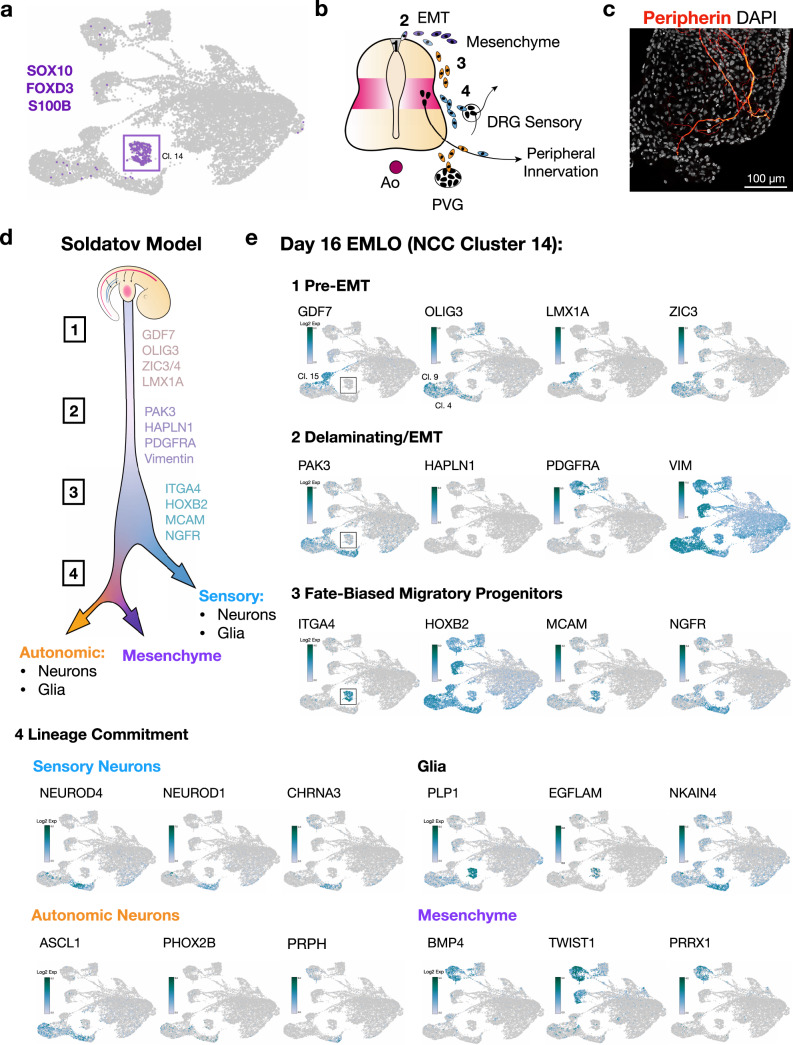
NCC trajectories mimic in vivo events in EMLOs. **a** Cluster 14 scRNAseq annotation as NCC by combined expression of SOX10, FOXD3, and S100B. **b** Schematic representation of neural tube cross-section depicts NCC in vivo developmental steps: pre-EMT (1), delamination/EMT (2), fate-biased lineage migration (3), and lineage commitment (4) to establish autonomic (yellow), sensory (blue), and mesenchymal (purple) anatomic derivatives^25^. DRG (dorsal root ganglia), PVG (prevertebral ganglia), Ao (aorta). **c** Peripheral neuron biomarker peripherin (red) in mesoderm–endoderm (ME) compartment (*N* = 2 repeat experiments). Individual scale bar is shown. **d** Updated NCC model by Soldatov et al.^24^ depicts developmental events described in **b** and the implicated genes. **e** Transcripts from the NCC processes labeled 1–4 as in **b**, **d** by scRNAseq in day 16 EMLOs (log_2_Exp). We infer that, in general, cells progress from the roof plate (cluster 15) and spinal cord (clusters 4, 9) as they undergo EMT to cluster 14 and express transcripts associated with fate-biased migration. Lineage-committed transcriptional programs are also detected for sensory and autonomic neurons, glia, and mesenchyme.

Gastruloids^6,8^ and NMOs^3^ have been shown to generate numerous cell types from more than one germ layer including NCCs. In vivo, competing transcriptional programs are first co-activated in NCCs, which are then lineage-biased in migrating progenitors^25^. We interrogated the diversity of cell types that arise in EMLOs and their relationship to the gut tube by scRNAseq and IF (Figs. 7, 8), focusing our analysis here on NCCs^3,26^. TFAP2α and SOX10 are canonical markers of NCCs^27^. We identified cluster 14 as the primary NCC cluster based on these and other canonical biomarkers (Figs. 7, 8, Supplementary Fig. 6). At day 6, SOX10 and TFAP2α largely overlapped by IF but were seen to partially diverge by day 20, where subpopulations of SOX10+ cells could be distinguished based on TFAP2α expression in the expanded cluster 14 (Fig. 7a, b). Similarly, a subpopulation of TFAP2α+ cells could be distinguished based on TUBB3 expression indicating both neuronal and non-neuronal NCC lineages (Fig. 7c), again validated both by scRNAseq and IF. A small subpopulation of EMLOs lacked the SC compartment (Fig. 5d). In these elongated ME-only aggregates, the gut tube was present (ISL1+), but the NCC biomarkers SOX10 and TFAP2α were distinctly lacking, suggesting that NCCs can populate the ME over time to produce neurons. However, a separate population of NCCs that can arise in ME given additional time or additional signaling manipulations is also supported by our data (Fig. 6d).

To assess epithelial–mesenchymal transition (EMT) that is thought to be a prerequisite for NCC migration, we performed IF of EMT biomarker, vimentin, in the context of extracellular matrix components Type I and Type IV collagen (Supplementary Fig. 6a). EMLOs exhibited a demarcated zone of EMT at the base of SC where bottlenecking of SC neurons occurs. By IF staining of PAX7 and PAX3, we validated that EMLO SC identity is largely that of dorsal-intermediate SC (PAX7), whereas PAX3 that marks NCCs originating in the SC were distributed throughout the ME (Supplementary Fig. 6b). As an internal control, non-elongated aggregates had dense, uniform PAX3 distribution. SOX10+ and TFAP2α+ cells were always observed in SC, interpenetrating between neuroectodermal rosettes, concentrated at the base of SC with only a subset observed in ME (Supplementary Fig. 6c).

N-cadherin, absent from the gut tube epithelium proper in EMLOs, was concentrated in SC but also adjacent to the distal anterior aspect of the gut tube, and the IF signal at this region increased as the number of cells populating ME and the ISL1+ ganglion increased (Fig. 7e–h)^24^. The proportion of EMLOs that exhibited a prominent peripheral ganglionic structure at day 22 was quantified in the three representative lines (Fig. 7i), indicating high reproducibility in patterning.

### Linking NCCs in EMLOs to in vivo events by scRNAseq analysis

Soldatov et al.^25^ recently described an updated model for NCCs in mouse based on extensive spatiotemporal sequencing analysis (Fig. 8b, d). In this model, NCC ontogeny is categorized into four stages that are: (1) pre-EMT (neural tube roof plate), (2) delamination/EMT, (3) fate-biased migration of progenitors, and (4) lineage commitment to sensory and autonomic peripheral neural cells and mesenchymal cell types. We applied this model that is based on sequential binary cell fate decisions to further characterize the NCCs in EMLOs and performed imaging of peripherin to detect peripheral neuronal axons in the ME compartment of day 40 EMLOs (Fig. 8c, e). A suite of genes implicated in pre-EMT NCCs located in the dorsal neural tube (GDF7, OLIG3, LMX1A, ZIC3) were seen expressed in cluster 15 that has a roof plate phenotype and clusters 4 and 9 that also have a neural tube/SC transcriptional phenotypes. By investigating the distribution of cells from pre-EMT through delamination/EMT (*PAK3*, *HAPLN1*, *PDGFRA*, *VIM*) to fate-biased migration (*ITGA4*, *HOXB2*, *MCAM*, *NGFR*), we observed more localization to cluster 14, providing indirect evidence of migratory NCC populations. Sets of genes for lineage commitment to sensory neurons, autonomic neurons, peripheral glial cells, and mesenchyme were also detected, thereby satisfying all four elements of the model^25^. The mesenchymal identity of the ME compartment was also identified as splanchnic mesoderm (*FOXF1*, *GATA4*, *GATA6*, *VIM*, *PDGFRA*) that is known to surround the epithelial structures in the trunk (Supplementary Fig. 7)^13^. Reciprocal signaling interactions between splanchnic mesenchyme and the gut tube are known to contribute to cardiogenesis and myogenesis^13,28^. We identified transcript and protein biomarkers of these processes in EMLOs (Supplementary Fig. 8–9). Axial trunk identity of NCCs is supported by *Hox* paralogous group analysis of cluster 14 including *HoxA3*, *HoxB3*, *HoxB5*, *HoxB9*, *HoxC6* (Supplementary Fig. 5c).

### EMLOs differentiate functional spinal neurons that express the MOR and have opioid-responsive firing

**Fig. 9:**
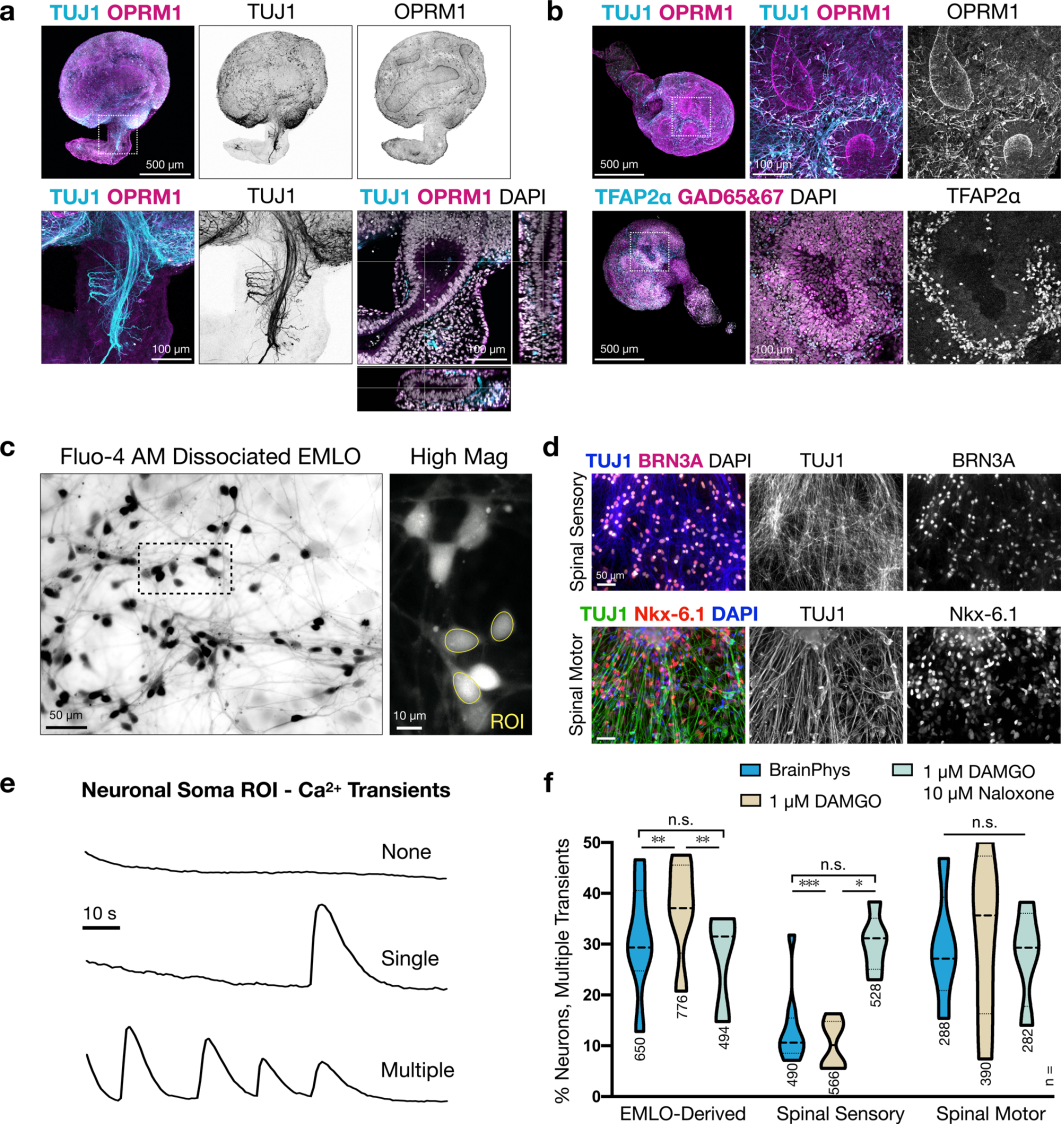
Modeling mu opioid receptor modulation in EMLO-derived neuronal cultures. **a** TUJ1 (cyan) and OPRM1 (magenta) IF in day 22 H3.3.1 EMLO. High magnification images provided (bottom-left) with multi-dimensional view of gut tube (bottom-right). **b** Top: OPRM1 expression in apical aspect of neural rosettes and basal neurons. Bottom: TFAP2α expression at the basal aspect of GABAergic rosettes. **c** Fluo-4 AM in dissociated EMLO cultures. High magnification image with example soma ROI (right). **d** 2D adherent spinal sensory neurons (top, TUJ1 in blue, BRN3A in magenta) and spinal motor neurons (bottom, TUJ1 in green, Nkx-6.1 in red) used as controls for opioid-responsive firing. IF of MOR was performed in *N* = 3 separate H3.3.1 EMLO formation experiments with similar results. **e** Examples of calcium transients quantified in dissociated EMLO neuronal soma using Fluo-4 AM. Shown are no calcium transients (none), one transient (single), or multiple transients (multiple) within a 1.5 min acquisition window. **f** Histogram of percent of neurons with multiple calcium transients. EMLO-dissociated cultures were compared with spinal sensory neurons (positive control) and spinal motor neurons (negative control). Baseline firing in BrainPhys (blue) was compared to addition of 1 μM DAMGO (tan), followed by 10 μM naloxone in the same cultures (light blue). *n* = number of neurons quantified. Statistical *p* values are as follows from left-to-right on the plot: **p = 0.0031, **p = 0.002, n.s. *p* = 0.766; ***p = 0.001, *p = 0.014, n.s. *p* = 0.972; n.s. *p* = 0.593, n.s. *p* = 0.64, n.s. *p* = 0.94). Violin plot statistics are as follows: EMLO-derived (BrainPhys: max = 47, min = 13, median = 29, q1 = 29, q3 = 39; BrainPhys + DAMGO: max = 32, min = 7, median = 11, q1 = 9, q3 = 15; BrainPhys + DAMGO + naloxone: max = 47, min = 32, median = 27, q1 = 21, q3 = 39); spinal sensory (BrainPhys: max = 48, min = 27, median = 37, q1 = 28, q3 = 46; BrainPhys + DAMGO: max = 16, min = 6, median = 10, q1 = 6, q3 = 14; BrainPhys + DAMGO + naloxone: max = 59, min = 7, median = 36, q1 = 19, q3 = 44); spinal motor (BrainPhys: max = 35, min = 15, median = 32; BrainPhys + DAMGO: max = 38, min = 23, median = 31, q1 = 25, q3 = 35; BrainPhys + DAMGO + naloxone: max = 38, min = 14, median = 29, q1 = 22, q3 = 34). Data reported as (mean ± s.e.m.). Individual scale bars provided.

There are few studies using stem cell models of opioid mechanisms yet opioids have important biomedical relevance. We applied EMLOs to calcium imaging studies to investigate the effect of MOR modulation on firing activity in various spinal neuron subtypes (Fig. 9). On day 22, EMLO staining for the MOR (or *OPRM1*) identified populations of OPRM1+ neurons and highlighted the apical aspect of neural rosettes (Fig. 9a, b top). OPRM1 signal was also distributed along the length of the gut tube lumen (Fig. 9a bottom). GABA-synthesizing enzymes GAD65&67 were expressed in SC in congruence with dorsal-intermediate SC identity and GABAergic neurotransmission that is required for the central antinociceptive effects of exogenous opioids (Fig. 9b bottom)^29,30^. We dissociated H3.3.1 EMLOs at day 22 using a neural tissue dissociation kit and formed 2D adherent neuronal networks (Fig. 9c). In parallel, we derived putative spinal sensory and motor neurons as positive and negative controls for MOR expression, respectively. Transcription factors BRN3A (sensory) and Nkx-6.1 (motor) were used to validate these neurons (Fig. 9d). 2D adherent neuronal differentiation was initiated on the same day as EMLO formation. Cultures were maintained for ten days after seeding in BrainPhys medium supplemented with BDNF, GDNF, and dibutyryl-cyclic AMP. On day 32, we performed successive calcium imaging experiments using Fluo-4 AM (Fig. 9e, f). Baseline calcium transients were first recorded in BrainPhys medium followed by 1 μM addition of the selective MOR agonist DAMGO in the same culture. Calcium imaging was repeated, followed by the addition of 10 μM of naloxone hydrochloride to outcompete DAMGO at MOR. The percentage of neuronal somata with multiple transients above the threshold over the duration of recordings (1.5 min) was quantified. In EMLO-derived cultures, the percentage of neurons with multiple transients was significantly reduced from 31 ± 5% (Supplementary Movie 4) to 13 ± 3% (Supplementary Movie 5) (mean ±s.e.m.). After naloxone addition, the percentage of neurons increased to 30 ± 5%. A similar result was observed in spinal sensory neurons, decreasing from 37 ± 4% to 10 ± 2%, and then increasing to 33 ± 7%. Spinal motor neurons did not show a statistically significant difference between conditions, although a slight percentage increase was observed after DAMGO addition. These data support the use of EMLOs as an early developmental model that may be informative to pharmacological studies with agents impacting many of the organs derived from the primitive structures that self-organize in the EMLO system.

## Discussion

In mammals, the breadth of development occurs in the post-implantation embryo, thereby limiting studies of early informative events required for a comprehensive understanding of human development and disease. Gastruloids offer an ability to study the co-development of interconnected systems and organogenesis that is not currently addressable by single-organoid methods. To date, gastruloid studies have primarily applied mouse ESCs to dissect polarized developmental processes with spatiotemporal accuracy^5,7–10^. To the best of our knowledge, no study using three germ-layer gastruloids has been used to investigate neurons specifically in mouse or human models, and, in particular, the neuronal trunk interactions with other primordial endoderm organ structures such as the primitive gut tube and enteric nervous system. The human multi-system EMLO gastruloid is therefore expected to be broadly useful for evaluating neurodevelopmental events impacting early CNS–PNS, endoderm, and neuromuscular co-emergent processes.

We developed the EMLO protocol as a tool for studying co-development of CNS and PNS neurons of the trunk. This approach modifies an existing protocol for neuromuscular trunk organoids (NMOs) that lack endoderm^3^. To accurately initiate developmental polarization events, the gastruloid literature emphasizes the importance of attention to cell number and paracrine signaling^5–7^, similar to the in vivo gastrula state. The NMO protocol applies 15–30× more cells for initial formation (~4500–9000 cells). Our strategy uses reduced initial cell numbers to achieve the desired length scale and more uniform exposure to external mitogens/morphogens for successful multi-lineage specification. NMP cellular starting material is less restricted by shortening the 2D induction with CHIR and FGF (Supplementary Fig. 1, 2). As well, we transition immediately to rotating culture to introduce additional mechanical cues at the critical early polarization stage. An important advance in our method is the ability to transition from a small multi-germ-layer gastruloid to larger organoid-like integrated structures with segregated compartments linked by neurons and the gut tube that follow developmental principles. This has not been achieved in any other gastruloid study, and is distinct from NMOs that were specifically designed to lack endoderm.

**Fig. 10:**
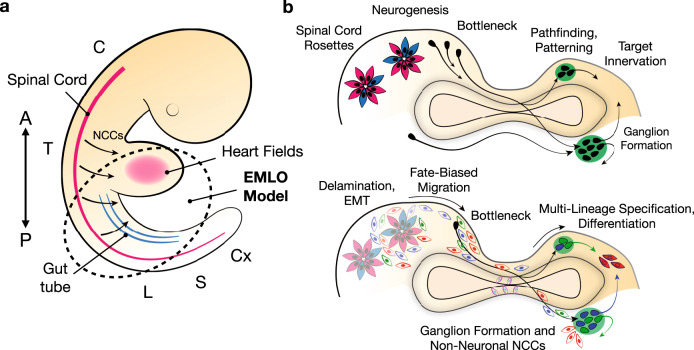
EMLO gastruloid models of human developmental events. **a** Schematic representation of human embryo. Cervical through coccygeal regions are labeled (C cervical, T thoracic, L lumbar, S sacral, Cx coccygeal). Primitive gut tube (blue) is shown in proximity to developing heart (pink) and spinal cord (red). Dotted circle represents the anatomic regions reflected in EMLO gastruloids and direction arrows indicate neural crest cell (NCC) migration. The anterior (A) and posterior (P) axis is shown. **b** Proposed model for neurogenesis, NCC migration and connectivity patterns in EMLOs including peripheral ganglion formation (green) and target innervation (top); proposed multi-lineage NCC behavior in EMLOs that parallels in vivo events^25^ (bottom). Neuroectodermal NCCs are shown in three colors (blue, red, green) to denote separate lineages during fate-biased migration, such as for vagal, trunk, and cardiac NCCs. Later-stage enteric neurons arising from endoderm are denoted in pink and blue.

Perhaps, the most remarkable feature that we demonstrate in EMLO gastruloids is the widespread marriage of developing neurons with the self-organizing primitive gut tube, reflective of the enteric nervous system (Figs. 2 and 5). Our data suggest a model (Fig. 10) in which the primitive gut tube acts as a morphological organizer of neuronal fibers. The term “organizer” as we use it here^28^ refers to regions of an embryo or gastruloid/organoid that can induce other cell fates and direct the pattern formation of other lineages. The temporal events in EMLO formation and high reproducibility are consistent with the gut tube acting as an early self-organizing event. In this regard, it is a bioenergetically prominent feature in EMLOs, and is inundated with neurons that are more compact around this structure versus elsewhere in the EMLO ME compartment (Supplementary Movie 3). Neuronal growth in tangential patterns is typically directed by gradient-driven responses, and we observe the consistent extension of multiple neuronal fibers around the gut tube and neuronal bodies that accumulate as ganglia. The stereotypic, dynamic transformation into neuronal assemblies with peripheral ganglionic structures that occurs in these EMLOs is reproducible. Based on our analysis, we favor the view that this represents a PNS-like region that is, in part, patterned by NCCs (Figs. 7, 8).

Ectodermal NCC ontogeny in development continues to be an exciting new area of discovery. In a recent comprehensive spatiotemporal sequencing study in mouse^25^, Soldatov et al. described a model for NCC ontogeny that differs from the classical model (Fig. 8). In this model, competing transcriptional programs are established in precursors even prior to EMT and are successively lineage-biased during migration to ultimately achieve a variety of differentiation landscapes and cell types in the periphery. The human EMLO data are consistent with this murine model (Figs. 7 and 8). By fixed time-series imaging and scRNAseq analysis, we infer that ectodermal NCCs originating in the SC compartment are able to populate the ME, effectively as proposed in Soldatov et al.^25^, by migrating and differentiating throughout the EMLO. Similar to in vivo events, early NCCs co-express lineage-specific transcriptional programs, but commit over time to diverge (Figs. 7 and 8). We summarize these points on ectodermal NCCs in a proposed EMLO model figure (Fig. 10). We have inferred ectodermal NCC migration in this study from analysis of multiple biomarkers in fully intact EMLOs and ME-only subpopulations, as well as the use of scRNAseq for detailed RNA expression profiling. Full validation of migration, however, will require the demonstration of live cell continuous motility. A truncated subset of EMLOs containing ME-only gut tube and mesenchyme does elongate, but lacks NCC biomarkers and TUJ1. These data are consistent with either migration of NCCs from SC into ME, or some contribution from the SC compartment for ME presence of non-ectodermal NCCs.

In the developing CNS, NMPs act at the major bifurcation point in generating the SC and somites^31^. In vitro, NMP-derived trunk NCCs^26,32^ have been demonstrated to differentiate into a variety of relevant PNS cell types, including autonomic sympathetic neurons^33,34^ and cells of the enteric nervous system^35^, as well as spinal motor neurons and interneurons of the CNS^16^. NMPs in EMLOs may constitute a transitory stage during the maturation of SC and ME compartments. Although EMLOs are formed with cell starting material co-expressing SOX2 and Bra that is characteristic of NMPs (Supplementary Fig. 2c–e), we no longer detect this population by these biomarkers at day 16 with scRNAseq. We favor the view that the SC region is patterned by NMPs, and that subsequent to initial early-stage polarization events, EMLO elongation is driven by mesenchymal and endodermal processes rather than by NMP-driven axial elongation. This is supported by the pharmacological inhibition of dual SMAD signaling pathways in developing EMLOs. All other gastruloid models require sustained NMPs with elongation, whereas, here we observe elongation via endoderm and mesoderm signaling.

Autonomic and enteric nervous systems form integrating neural circuitry with communicating nodes called ganglia in which neuronal cell bodies reside. In EMLOs, highly reproducible ganglionic structures emerge at the base of the gut tube and are comprised of peripheral neurons with NCC-derived expression profiles. By scRNAseq of day 16 EMLOs, we identified *TUBB3*+ neurons co-expressing *ASCL1* and *PHOX2B,* indicating the presence of developing autonomic neurons. Future investigations into the emergence and progressive distribution and organization of autonomic neurons using techniques to simultaneously apply scRNAseq with cell organization as the EMLOs mature will be informative, including the identification of non-ectodermal derived enteric neurons^24,36^. This, in addition to the ectodermal origin of NCCs, supports the possibility of two separate neural lineages for the enteric nervous system^24^. As well, it was recently demonstrated in murine models that cranial ectodermal NCCs undergo an in vivo reprogramming event wherein pluripotency genes *OCT4* and *Nanog* are reactivated prior to non-ectodermal NCC lineage commitment^37^, further emphasizing the complexity of NCC and trunk neuronal ontogeny. The EMLO model recapitulates dual NCC ectodermal and endodermal embryonic origins of neurons contributing to the human enteric nervous system that was recently described in mice (Fig. 6c, d)^24^. On day 16 scRNAseq data, we identified a subpopulation of *FOXA2*/*EPCAM*/*SOX17* endodermal cells that also co-express neuronal *TUBB3* and *MAP2* transcripts. It is worth noting that a large number of developmental regulators are now known to be co-involved in both nervous system and gastrointestinal development such as *MNX1*/HB9, *ISL1*, *CDX2*, *SOX2*, *PAX6*, *FOXA1*, *FOXA2*, *NKX-6.1*, *ALDH1A2*, *SHH*, *WNT5A*, *BMP4,* and *Hox* genes to name several. Human EMLOs may therefore constitute the only current human system capable of enabling the further investigation of ectodermal and non-ectodermal neuronal origins of the enteric nervous system for validation.

Human model systems that might be useful towards understanding the early neurodevelopmental impacts of pharmacologic agents, such as opioids used biomedically, are limited. Very few studies in the literature apply human stem cell models to opioid investigations, and virtually none that include organoids or diverse genotypes^38–40^. Dorsal lamina GABAergic interneurons have an essential role in opioid-induced central antinociception^30^, and this regional identity is present in EMLOs by default (Fig. 9). We chose to evaluate the MOR and opioid biomimetic compounds owing to their relevance to the trunk and SC development^29^, although their importance encompasses broader CNS neuronal plasticity and biomedical uses^41^. This analysis is limited by the use of EMLO and dissociated neurons to allow specific neuronal maturation for quantitative comparison of motor and sensory neurons using calcium imaging (Fig. 9). It is proof-of-principle of the potential of EMLOs for modeling neurogenic changes of clinical and pharmacological relevance in regard to integrated CNS, PNS, and other organ systems that have self-organizing precursors in EMLOs. In future studies, the ability to take full advantage of developmental insights from EMLOs or other gastruloid, organoid, and assembloid models is expected as the technology for detailed analysis of 3D structures continues to advance. The rapid pace of signaling events, the resolution needed to capture those events in thick samples, and the need for quantification supports the mixed analysis of both dissociated and intact organoids. The expression of MOR receptors in the membrane in EMLOs and our 2D analysis have not previously been described in the field since human stem cell models are not yet well-established for opioid studies.

In this work, we have not yet explored all optimized outcomes for EMLOs. For example, in earlier work^12^, we showed that different ED-iPSC lines can achieve uniform high-efficiency cardiomyocyte differentiation and contractility when we optimize the CHIR 99021 concentration^12^. This was beneficial when analyzing clonal replicate lines derived from the same donor (e.g., H3.3.1 vs. H3.1.1). The need for optimization was similarly observed by other researchers, such as in the anteroposterior gastruloid study using hESCs^6^, and for line-specific differences to generate NMOs^3^. In this current work, we apply a single protocol to nine lines that successfully generate EMLOs. At last, we were unable to identify commercially available gut tube-specific inhibitors. We recognize that dual SMAD inhibition can inhibit both mesoderm and endoderm, and therefore enhance commitment to neuroectodermal lineages, and so future work will be necessary to more fully elucidate the role of the gut tube as a developmental organizer using specific gut inhibitors.

Together, EMLOs constitute an important bridge to more systemic tissue interactions and innervation studies together with other gastruloid and assembloid models^42,43^ (Fig. 10). The human EMLO system enables the continued dissection of early neurodevelopment and, importantly, continued differentiation within an embryo-like, multi-lineage context. We are also exploring EMLOs in regard to skeletal muscle differentiation and optimization for heart field progression (Supplementary Figs. 8–9). The human spine is integral to CNS–PNS functions and EMLOs are expected to also provide an ability to investigate mechanisms for impaired human CNS neuronal regeneration with respect to peripheral neurons that has direct relevance for elucidating innate mechanisms preventing SC injury repair as well as for identifying new human therapeutics. Our research with ED-hiPSC lines also expands stem cell research multi-ethnic representation to more accurately reflect the diverse US population. As developmentally relevant human models, EMLOs are expected to offer substantial insights in modeling organogenesis, systems interconnectivity, and innervation in a multi-lineage platform.

## Methods

### Human ED-hiPSC lines and culture conditions

Nine ED-hiPSC lines were previously generated in a Paluh laboratory study under an approved ISSCR protocol using commercial de-identified fibroblast cell lines of three individuals obtained from Coriell11 and were extensively characterized by our laboratory 12, 15, 16. Coriell cell lines were obtained under informed consent according to the company website. Catalog numbers are as follows: GM22268 (F3; African American male, foreskin, 1 day); AG08498 (A-2; Asian male, foreskin, 1 day); GM22186 (H3; Hispanic-Latino male, foreskin, 1 day). hiPSC lines were validated for normal karyotype. Details of ED-hiPSC characterization are provided in Supplementary Table 1. hiPSCs were maintained in pluripotency medium mTeSR Plus (STEMCELL Technologies) supplemented with 1× penicillin–streptomycin (P–S) on hESC-qualified Matrigel (1:100 dilution; Corning) at 37°C, 5% CO2. Cultures were passaged 1:6 in six-well plates every 4–7 days using Gentle Cell Dissociation Reagent and cryopreserved in mFreSR according to manufacturer’s instructions (STEMCELL Technologies). All lines were expanded and stored at a low passage number.

### EMLO gastruloid formation and signal manipulation

Our gastruloid protocol begins with the pretreatment of 2D adherent hiPSC colonies maintained in mTeSR Plus pluripotency medium. At ~60% confluency, mTeSR Plus was replaced with the N2B27 basal medium supplemented with 3 μM CHIR 99021 (Tocris Bioscience) and 40 ng/ml basic fibroblast growth factor bFGF/FGF2 (R&D Systems). N2B27 basal medium: 1:1 Dulbecco’s Modified Eagle Medium (DMEM)/F-12:neurobasal plus medium, 2% (v/v) B27 Plus supplement, 1% (v/v) N2 supplement, 1× GlutaMAX, 1× MEM non-essential amino acids, 1× P–S. Colonies were maintained in pretreatment medium for 2 days, with fresh culture medium replenished each day. On the day of dissociation (day 0), wherein migratory cells became visible at the colony edge, cells were dissociated with 1:1 accutase:HBSS (Hank’s Balanced Salt Solution) (Ca-Mg free) at 37°C for 5 min followed by manual trituration with a P-1000 pipette. Six-well plates were pretreated with anti-adherence rinsing solution (STEMCELL Technologies) for 5 min incubation at room temperature followed by two rinses with equal volumes of fresh HBSS. Cells were suspended in N2B27 supplemented with 10 ng/ml FGF2, 2 ng/ml IGF-1, 2 ng/ml HGF (R&D Systems), and 50 μM Y-27632 (Tocris Bioscience), and single-cell suspensions were distributed at 2 × 10^6^ cells/ml in 2 ml per well. Well plates were transitioned to shaking suspension culture using an orbital shaker at 75 rpm clockwise in a humidified incubator. The next day, one-half volume of medium was replaced with fresh medium N2B27 supplemented with 2 ng/ml IGF-1, 2 ng/ml HGF. On day 3, the entire volume of medium was replenished and shaking cultures were maintained to day 4. On day 4, individual wells were transferred 1:1 to 100 mm petri dishes pretreated with anti-adherence rinsing solution in fresh N2B27 only (7–8 ml per dish) and maintained at 70 rpm. One-half volume of fresh N2B27 was replenished every 3–4 days. EMLO gastruloids were maintained at least to day 22, and in some cases to day 40.

To test the effect of dual SMAD inhibition on ME compartment morphology, 200 nM LDN 193189 and 10 μM SB 431542 (Tocris Bioscience) was added during a critical endoderm expansion/elongation period between days 2 and 10. LDN and SB were excluded after day 10 and EMLOs were quantified at day 22. Dorsal–ventral regional identity of spinal domains was manipulated by addition of 500 nM Hh-Ag1.5 (Cellagen Technology) on day 10 after elongation had occurred and maintained to day 22 for imaging and quantification by cell counting.

### Spinal sensory and motor neuron derivation

Spinal sensory neurons^44^ and spinal motor neurons^15,16^ were derived similarly to as previously described and detailed as follows. For nociceptor sensory neurons, hiPSCs were seeded onto freshly-coated Matrigel 20,000–40,000 cells/cm^2^ in mTeSR Plus with 10 μM Y-27632. When cells were confluent (day 1 of differentiation), the medium was changed to knockout DMEM with 15% (v/v) knockout serum replacement, 1× GlutaMAX, 1× MEM non-essential amino acids, 1× P–S. Dual SMAD inhibition was achieved by 100 nM LDN 193189 and 10 μM SB 431542 days 0–5 and nociceptors were induced by addition of 3 μM CHIR 99021, 10 μM SU 5402, 10 μM DAPT between days 2 and 10. Beginning at day 4, N2 supplement was added every other day at 25% increments to day 10. At day 20, cultures were maintained in BrainPhys supplemented with N2/B27 and 10 ng/ml BDNF, 10 ng/ml GDNF (R&D Systems), 1 μM dibutyryl-cyclic AMP (dbcAMP).

For spinal motor neurons, restricted NMPs were induced in 2D adherent hiPSC colonies (~60% confluency) by culture in N2B27 supplemented with 40 ng/ml FGF2, 40 ng/ml FGF8 (R&D Systems), 2 μM CHIR 99021, 10 μM DAPT (Tocris Bioscience), 10 μM SB 431542, 100 nM LDN 193189, 0.36 U/ml heparin^15,16^ (Millipore Sigma). The medium was changed daily for four days. On day 4, NMPs were split in N2B27 supplemented with 100 nM RA, 200 nM Hh-Ag1.5 and maintained to day 20 at which point, BrainPhys supplemented medium described above was added. Spinal sensory and spinal motor neuron differentiation began the same day as EMLO formation. Day 22 cultures were passaged to chambered coverglass and cultured for 10 days prior to calcium imaging.

### Phase-contrast imaging, whole-mount IF, and tissue clearing

For phase-contrast microscopy, samples were imaged at room temperature directly in culture plates. Images were acquired using a Zeiss Invertoskop 40 C (5×/0.12 CP-Apochromat, 10×/0.25 Ph1 A-Plan, and 20×/0.30 Ph1 LD A-Plan, 40x/0.50 Ph2 LD A-Plan) mounted with an Olympus DP22 color camera and cellSens acquisition software. Whole-mount IF preparation was performed as previously described and detailed as follows^9^. EMLOs were pooled on the day of fixation, rinsed once with 1× phosphate-buffered saline (PBS), and fixed in 10% neutral-buffered formalin solution at 4°C for 2 h. EMLOs were rinsed three times in 1× PBS for 5 min. Samples were then permeabilized by three successive incubations in 0.2% Triton X-100 in 1× PBS (PBST) for 20 min at 4°C, and blocked overnight in 1% bovine serum albumin (BSA) in PBST. The next day, samples were distributed to 12-well plates in 1 ml blocking solution per well. Primary antibodies were added to requisite dilutions (see Supplementary Information). Plates were left rocking at 4°C for 48–72 h, then rinsed three times in blocking solution followed by three times in PBST for 5 min at room temperature in 2 ml centrifuge tubes. Species-matched secondary antibodies were incubated 1:1,000 with additional NucBlue fixed cell stain (Invitrogen) directly in 2 ml centrifuge tubes, rocking overnight at 4°C. For triple-antibody stains, goat anti-mouse or goat anti-rabbit Cy5 secondary antibodies were added the next day after removal of donkey anti-goat AlexaFluor secondary antibody by additional wash steps. All excess secondary antibodies were ultimately removed by two wash steps in blocking solution followed by two wash steps in PBST. Stained and rinsed EMLO samples were equilibrated in 0.1 M phosphate buffer (PB: 0.025 M NaH_2_PO_4_, 0.075 M Na_2_HPO_4_, pH 7.4) by three successive incubations of 5 min at room temperature. EMLOs were then post-fixed in 10% neutral-buffered formalin for 20 min at 4°C, and rinsed three more times in 0.02 M PB. To clear samples, PB was aspirated and replaced with 200 μl of 88% Histodenz solution (w/v) in 0.02 M PB. Samples were left in the dark at 4°C for 24–48 h and then mounted on glass slides, sealed in clear nail polish.

Primary antibodies are listed with company, catalog number, and RRID in Supplementary Data 1. The following antibody dilutions in 1% BSA (1× PBS) were used for the corresponding antibodies: anti-SSEA3 (4 μg/ml); anti-SSEA4 (4 μg/ml); anti-SOX2 (mouse, 10 μg/ml); anti-SOX2 (goat, 5 μg/ml); anti-brachyury (5 μg/ml); anti-GATA4 (5 μg/ml); anti-GATA6 (5 μg/ml); anti-CDX2 (2 μg/ml); anti-ISL1 (2 μg/ml); anti-TFAP2A (5 μg/ml); anti-SOX10 (5 μg/ml); anti-Collagen IV α1 (10 μg/ml); anti-collagen I α1 (1:500); anti-Vimentin (5 μg/ml); anti-CDH1/E-cadherin (5 μg/ml); anti-FOXA2 (5 μg/ml); anti-FOXF1 (1:2,000); anti-SOX17 (5 μg/ml); anti-β-III-tubulin (rabbit, 1:2,000); anti-β-III-tubulin (mouse, 1:1,000); SMI312 cocktail (1:1,000); anti-Peripherin (1:200); CDH2/N-cadherin (1:200); anti-ZO-1 (1:1,000); anti-OPRM1 (1:500); anti-GAD65&67 (1:1,000); anti-TLX3 (1:1,000); anti-LBX1 (2 μg/ml); anti-PAX2 (5 μg/ml); anti-PAX3 (5 μg/ml); anti-PAX7 (10 μg/ml); anti-LXH9 (2 μg/ml); anti-CHX10 (1:500); anti-NKX-6.1 (0.5 μg/ml); anti-BRN3A (1:1,000); anti-BMP2/4 (5 μg/ml); anti-desmin (5 μg/ml); anti-Myosin Heavy Chain (10 μg/ml); anti-cardiac troponin-T (MAB1874). All secondary antibodies were incubated at 1:1000 in the same buffer.

Samples were imaged on a Leica confocal TCS SP5 II system in conjunction with Leica Application Suite Advanced Fluorescence software (v2.7.3.9723). The SP5 II system was equipped with 10×/0.30 HCX PL FLUOTAR air, 20×/0.70 HC PL APO CS air or immersion, and 40×/1.25 HCX PL APO immersion objective lenses. Complete or partial Z-stacks were acquired at ~2 μm separation distance.

### EMLO single-cell dissociation by cold activated protease for scRNAseq

Day 16 EMLOs were dissociated to single cells using a cold activated protease method previously described^45^ and essential details are provided here. Dissociation solution was comprised of 10 mg/ml psychrophilic *Bacillus licheniformis* (Creative Enzymes) protease and 125 U/ml DNase (Millipore Sigma) in ice-cold 1× PBS supplemented with 5 mM CaCl_2_. In all, ~35 EMLOs were pooled into a 2 ml centrifuge tube and allowed to settle. Culture medium was aspirated and 1 ml ice-cold dissociation solution was added to EMLOs and incubated on ice. Every 30–60 s, EMLOs were triturated with a P-1000 pipette and returned to ice. After 15 min, single-cell suspension was validated by optical inspection. 1 ml of ice-cold 1× PBS with 10% fetal bovine serum (FBS) was added and cells were pelleted by centrifugation at 1200 × *g* for 5 min. The supernatant was completely aspirated. Cells were resuspended in 1× PBS/10% FBS and counted, centrifuged once more, aspirated completely and resuspended to ~1 × 10^6^ cells/ml in CryoStor CS10 cryopreservation medium (BioLife Solutions) that was filtered through a 40 μm cell strainer and then added to a 1.8 ml Nunc cryo-storage tube. Cells were frozen at −80 °C and shipped overnight on dry ice to University of Buffalo Genomics and Bioinformatics Core.

### Single-cell sequencing and cluster annotation

Samples received by the University of Buffalo Genomics and Bioinformatics Core were immediately stored at −80 °C. On the day of cell capture, the sample was thawed in a 37°C water bath for 2 min with gentle shaking. The cells were transferred to a 15 ml tube and prewarmed media (RPMI1640+ 10% FBS) was added by dropwise dilution to a final volume of 10 ml. Cells were spun at 300 × *g* for 5 min. This washing procedure was performed three times in total. After the final spin, the dissociated cell pellet was resuspended in 500 μl media and counted using a Logos Biosystems LUNA II in bright field mode (0.4% trypan blue exclusion). Single cells were loaded onto a 10× Genomics Chromium platform using the NextGem V3.1 single-cell reagent kit according to the manufacturer’s instructions. cDNAs were generated from captured transcripts. After cell capture and cDNA preparation, the cDNA product was used to generate Illumina-ready double-stranded DNA libraries. The concentration of the libraries was determined by the Agilent Fragment Analyzer and the Roche KAPA universal qPCR kit. Samples were diluted to 250 pM and denatured for sequencing on the NovaSeq 6000 (28 × 91). Upon sequencing run completion, the sample was demultiplexed using 10× Genomics Cell Ranger analysis software (V4.0.0), which associates each transcript to its cell-of-origin and produces sequencing quality reports, fastq files, and per-cell-per-transcript count matrices. The data were reviewed for quality to ensure sufficient mean-reads per cell (64,538), a high fraction of reads in cell (80.6%), and confident mapping rates to the Grch38 reference genome (91.6%). Data were then analyzed using the 10× Genomics Loupe Browser (V5.0.0). Clusters were annotated based on canonical biomarkers, provided here as a supplemental file (Source data file). Rossi et al.^10^ provide another comprehensive list.

### EMLO dissociation for 2D neuronal cultures and IF

Day 22 EMLOs (~25 count) were pooled and dissociated using the Neural Tissue Dissociation Kit (P, palpain) according to manufacturer’s instructions (Miltenyi Biotec) and similarly to a previous report^46^. Samples were manually triturated with a P-1000 pipette. Dissociation was confirmed by visual inspection. Spinal sensory and spinal motor neurons used as controls for calcium imaging were derived as detailed above. On day 22 in differentiation, adherent cultures were detached with 1:1 accutase in HBSS and seeded into chambered wells of matrigel-coated coverglass. At the same time, day 22 dissociated EMLOs were seeded similarly. Parallel cultures were maintained in BrainPhys supplemented with N2, B27, and 10 ng/ml BDNF, 10 ng/ml GDNF, 1 μM dbcAMP. One-half volume of media was replenished every 3–4 days.

For IF of adherent spinal sensory and motor neurons, day 32 adherent cultures were rinsed once in 1× PBS and fixed with 10% neutral-buffered formalin for 30 min at 37°C. Samples were rinsed again in 1× PBS, permeabilized for 5 min in 0.1% Triton X-100, and blocked for 30 min in 1% BSA fraction V (1× PBS) at room temperature. Primary antibodies were applied in 1 ml fresh blocking buffer and incubated at 4°C overnight. Samples were rinsed thoroughly in 1× PBS before applying immunoglobulin- and species-matched secondary antibodies for 1 h in the dark with NucBlue fixed cell stain (4°C). Cells were imaged directly in chambered coverglass wells. Wide-field fluorescence microscopy was performed using a Zeiss Axio Observer.Z1 inverted fluorescence microscope (×20/0.8 air and ×63/1.4 oil Plan-Apochromat DIC objectives). Images were acquired using a Hamamatsu ORCA ER CCD camera and Zeiss AxiovisionRel software (ver. 4.8.2). For adherent cultures, 10–20-slice Z-stacks were gathered at 1 µm separation distance and compressed using the extended focus feature. If necessary, images were adjusted linearly for brightness in Keynote or ImageJ.

### MOR modulation and calcium imaging

On the day of live cell calcium imaging, 50 µg Fluo-4 AM cell-permeant dye (Invitrogen) was diluted in 10 µl of 20% pluronic F-127 in DMSO. Fluo-4 AM was diluted 1:1000 in BrainPhys medium and added to coverglass chamber wells after rinsing cells once in HBSS. Samples were incubated at 37°C for 30 min. After incubation, Fluo-4 AM medium was removed, washed once in HBSS and fresh BrainPhys without phenol red was added. Samples were imaged directly on the Zeiss Axio Observer.Z1 system. Time-lapse series were acquired at 50 ms exposure using a 488 nm LED at 200 ms intervals for 1–1.5 min duration. After imaging baseline activity in BrainPhys, 1 μM DAMGO (MOR agonist, 10 mM stock in water; Abcam) was added directly to chamber wells and imaged again. The competitive opioid receptor antagonist naloxone hydrochloride was then added at 10 μM (10 mM stock in water; Abcam). EMLO-dissociated cultures, spinal sensory, and spinal motor neuron samples were imaged in succession to control for time of Fluo-4 AM cell loading. Time-lapse series were quantified in ImageJ.

### Neuromuscular organoids, cerebral organoids, neurospheres, and small molecule manipulation

The protocol for previously reported polarized NMOs^3^ was validated using ED-hiPSC lines (Supplementary Fig. 1). In brief, hiPSC colonies were dissociated with accutase and seeded onto matrigel as single cells at 100,000/cm^2^ in N2B27 supplemented with 3 μM CHIR, 40 ng/ml FGF2. The medium was changed every day for 3 days. At day −3, uniform NMps were dissociated with accutase and forced to aggregate in 96-well plates (9–10,000 cells/well). At the time of dissociation, the culture medium was changed to N2B27 supplemented with 10 ng/ml FGF2, 2 ng/ml HGF, 2 ng/ml IGF-1 (100 μl/well). In all, 50 μM ROCK inhibitor was used at time of aggregation. One-half volume of medium was replaced with 100 μl N2B27 supplemented with 2 ng/ml HGF and IGF-1 24 h post aggregation. After day 4, aggregates were maintained in N2B27 only. Day 10 organoids were transferred to a 60 mm dish and moved to an orbital shaker (70 rpm).

To test the effect of RA on EMLO elongation, 1 μM RA was added to the shaking culture medium on day 2 of EMLO formation and maintained to day 13. In a separate experiment, we converted 2D adherent cultures that were pretreated for three days with 3 μM CHIR 99021 and 40 ng/ml FGF2 to spinal cord neural stem cells (scNSCs) by addition of 100 nM RA and 200 nM of the potent Hedgehog agonist Hh-Ag1.5. After 10 days, neurospheres were generated from adherent scNSCs on the orbital shaker at 75 rpm and were monitored for elongation.

Cerebral organoids were formed using a modified protocol from Trujillo et al.^47^ combined with the STEMCELL Technologies STEMdiff Cerebral Organoid and Maturation kit. In brief, ~70% confluent hiPSC colonies maintained in mTeSR Plus were dissociated with Accutase diluted 1:1 in HBSS and transferred to 6-well plates treated with anti-adherence rinsing solution (STEMCELL Technologies) at ~4 × 10^6^ cells per well. Cells were kept shaking at 95 rpm in mTeSR Plus supplemented with 1 μM dorsomorphin (Tocris Bioscience) and 10 μM SB 431542 (Tocris Bioscience). In brief, 5 μM ROCK inhibitor was initially used. At day 3, the medium was changed to N2B27 with the same small molecule and maintained for 7 days to achieve neural induction. We then switched neural expansion medium (STEMCELL Technologies) for 7 additional days, followed by maturation medium (STEMCELL Technologies), and maintained to day 50.

### Human stem cells ethics statement

No new cell lines were generated in this study. The ED-hiPSC lines used are published and were previously generated in a Paluh laboratory study under an approved ISSCR protocol using commercial de-identified fibroblast cell lines of three individuals obtained from Coriell^11^. The Coriell cell lines were obtained under informed consent according to the company website.

### Statistical analysis and reproducibility

The detailed protocol for EMLO formation is publicly available^48^. Raw data were compiled in Microsoft Excel (v16.16.27) and exported to GraphPad Prism (v9.0.0) for plotting and statistical analysis. Data are reported as (mean ± s.e.m.) and analyzed using an unpaired two-tailed *t* test. EMLO cell nuclei were manually counted using ImageJ to quantify IF data using Z-slices at least 20 μm apart to avoid double counting. ****p < 0.0001, ***p < 0.001, **p < 0.01, *p < 0.05, n.s. not significant (*α* = 0.05). Power analysis was not performed. Detailed information for each experiment is provided in Results and Figure Legends. Key resources including primary antibodies, chemicals, and other reagents, software, equipment, and commercial kits are provided (Supplementary Data 1), along with the Source date file and scRNAseq gene annotation lists (Supplementary Data 2).

### Reporting summary

Further information on research design is available in the Nature Research Reporting Summary linked to this article.

## Supplementary information

Supplementary Information

Supplementary Data 1

Supplementary Data 2

Supplementary Movie 1

Supplementary Movie 2

Supplementary Movie 3

Supplementary Movie 4

Supplementary Movie 5

Reporting Summary

Description of Additional Supplementary Files

## Data Availability

The authors declare that all data supporting the findings of this study are available within the article and its Supplementary Information files or from the corresponding author upon reasonable request. The raw single-cell sequencing data have been deposited in the Gene Expression Omnibus (GEO) database under accession code: GSE166603. Figures for this manuscript were made in Keynote (v10.3.5) and Adobe Illustrator Creative Commons 2020. Data plots were generated using GraphPad Prism 9. Source data are provided with this paper.

## Source data

Source Data
