# Supplementary material for: Co-development of central and peripheral neurons with trunk mesendoderm in human elongating multi-lineage organized gastruloids
Source: Nat Commun. 2021 May 21;12:3020. doi: 10.1038/s41467-021-23294-7 (PMC8140076; doi:10.1038/s41467-021-23294-7)
Supplement: Supplementary file 1 — Supplementary Information [file 41467_2021_23294_MOESM1_ESM.pdf]

SUPPLEMENTARY INFORMATION

Co-development of central and peripheral neurons with trunk mesendoderm in human elongating multi-lineage organized gastruloids

Zachary T. Olmsted[1] and Janet L. Paluh[1]

[1]State University of New York Polytechnic Institute, Colleges of Nanoscale Science and Engineering, Nanobioscience Constellation, Albany, NY 12203

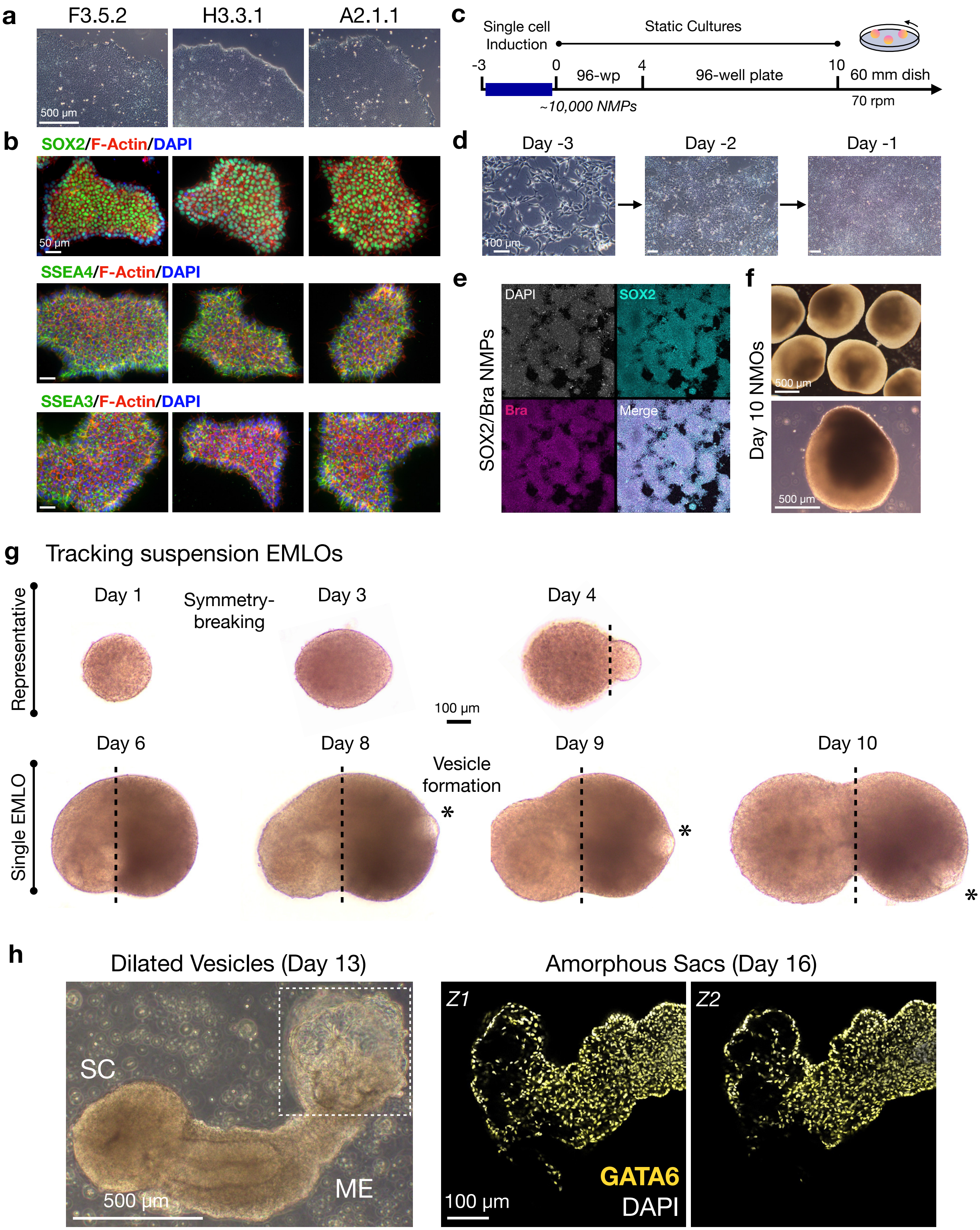
a
F3.5.2
H3.3.1
A2.1.1
500 µm
b
SOX2/F-Actin/DAPI
50 µm
SSEA4/F-Actin/DAPI
SSEA3/F-Actin/DAPI
c
Single cell
Induction
Static Cultures
-3
0
96-wp
4
96-well plate
10
60 mm dish
~10,000 NMPs
70 rpm
d
Day -3
Day -2
Day -1
100 µm
e
SOX2/Bra NMPs
DAPI
SOX2
Bra
Merge
f
Day 10 NMOs
500 µm
500 µm
g
Tracking suspension EMLOs
Representative
Day 1
Symmetry-
breaking
Day 3
Day 4
100 µm
Single EMLO
Day 6
Day 8
Vesicle
formation
Day 9
Day 10
*
*
*
h
Dilated Vesicles (Day 13)
SC
ME
500 µm
Amorphous Sacs (Day 16)
Z1
Z2
GATA6
DAPI
100 µm

Supplementary Fig. 1 Induction and tracking of pluripotent ED-iPSC representative lines to neuromuscular trunk organoids and EMLO gastruloids. a, Phase contrast images of low passage number F3.5.2, H3.3.1, A2.1.1 pluripotent hiPSC colonies. **b**, Pluripotency markers SOX2, SSEA4 and SSEA3. **c**, Summary of neuromuscular trunk organoid protocol[1]. Stem cell colonies were stained for IF in N = 1 experiment. **d**, Single cell NMP induction for three days. **e**, Rich SOX2+/Bra+ NMP population after three days of single cell CHIR/FGF2 induction in N2B27. **f**, Day 10 neuromuscular trunk organoids (NMOs) after transition to 60 mm dish. **g**, Representative early (top) and single-EMLO tracking (bottom) by phase contrast microscopy. Asterisk (*) indicates vesicle formation. NMOs were formed and imaged by IF in N = 2 repeat experiments. **h**, Large dilated anterior vesicles in day 13 EMLO (left) that later appear as amorphous sacs (day 16) lined by GATA6 cells (right images). This feature of EMLOs was observed in all N = 11 separate formation experiments. Individual scale bars provided.

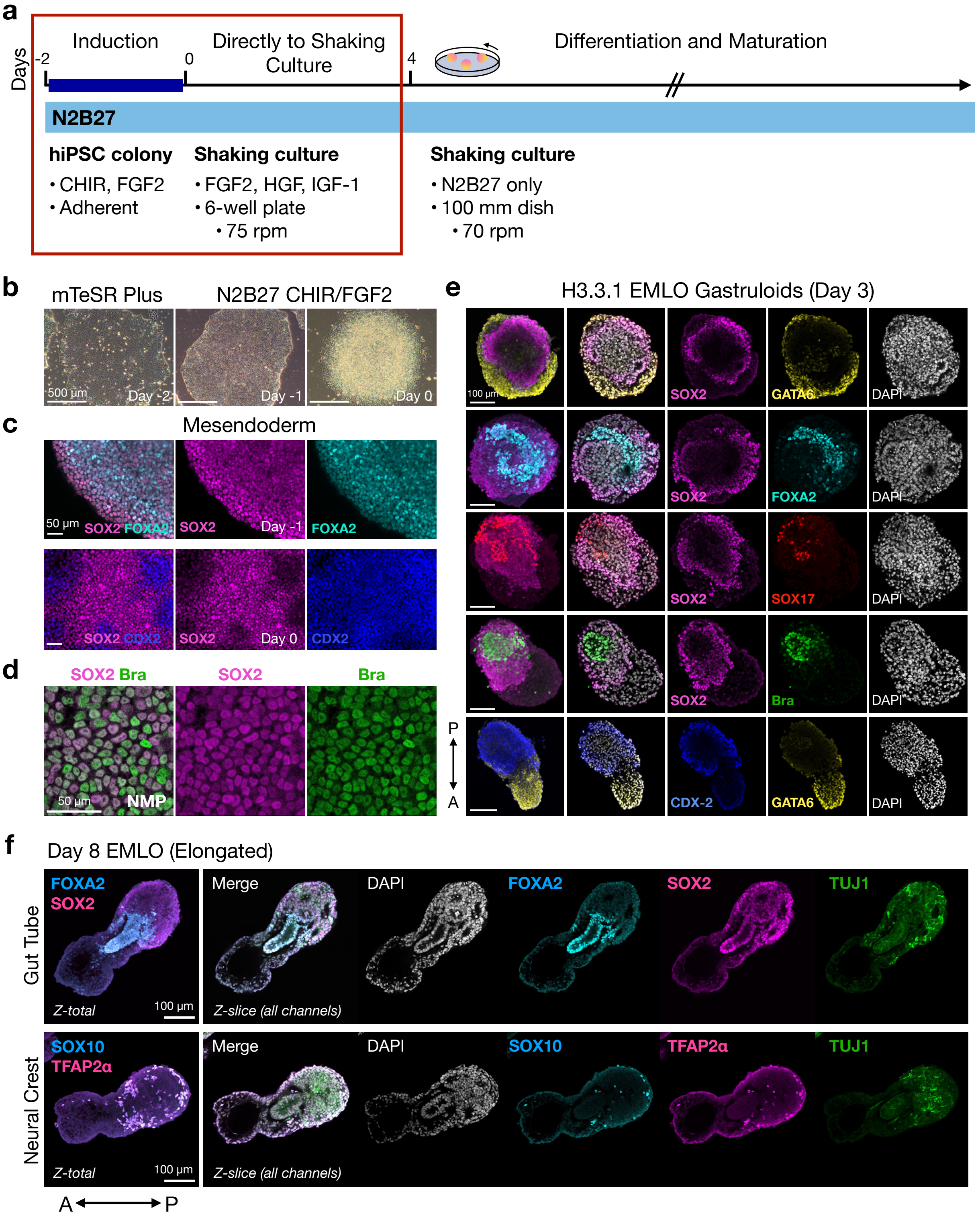
a
Days
Induction
Directly to Shaking Culture
Differentiation and Maturation
-2
0
4
N2B27
hiPSC colony
• CHIR, FGF2
• Adherent
Shaking culture
• FGF2, HGF, IGF-1
• 6-well plate
• 75 rpm
Shaking culture
• N2B27 only
• 100 mm dish
• 70 rpm
b
mTeSR Plus
N2B27 CHIR/FGF2
500 µm
Day -2
Day -1
Day 0
c
Mesendoderm
50 µm
SOX2 FOXA2
SOX2
Day -1
FOXA2
SOX2 CDX2
SOX2
Day 0
CDX2
d
SOX2 Bra
SOX2
Bra
50 µm
NMP
e
H3.3.1 EMLO Gastruloids (Day 3)
100 µm
SOX2
GATA6
DAPI
SOX2
FOXA2
DAPI
SOX2
SOX17
DAPI
SOX2
Bra
DAPI
P
A
CDX-2
GATA6
DAPI
f
Day 8 EMLO (Elongated)
Gut Tube
FOXA2
SOX2
Z-total
100 µm
Merge
Z-slice (all channels)
DAPI
FOXA2
SOX2
TUJ1
Neural Crest
SOX10
TFAP2α
Z-total
100 µm
Merge
Z-slice (all channels)
DAPI
SOX10
TFAP2α
TUJ1
A
P

Supplementary Fig. 2 Short-term 2D induction of EMLO starting material and biomarker analysis during early polarization. **a**, EMLO differentiation protocol with day -2 to day 3 highlighted. **b**, H3.3.1 hiPSC colony in mTeSR Plus medium versus N2B27 + CHIR/FGF2 prior to dissociation. These images are representative of each EMLO formation experiment (N = 11). **c**, IF of SOX2 and mesendoderm marker FOXA2 after one day of pretreatment (top) and SOX2/CDX2 after two days of pretreatment (bottom). **d**, High magnification IF depicts SOX2/Bra co-localization after one day of pretreatment. N = 2 biological repeat experiments for IF. **e**, Expression and compartmentalization of multi-lineage biomarkers in day 3 aggregates. SOX2, GATA6, FOXA2, SOX17, Brachyury (T/Bra), and CDX2 are shown. **f**, Primitive gut tube formation and early NCCs in elongated day 8 EMLO. FOXA2/SOX2/TUJ1 (top) and SOX10/TFAP2α/TUJ1 (bottom) Z-slices are shown with DAPI. Leftmost image is maximally-projected Z-stack (no TUJ1 or DAPI). IF of day 3 and day 8 EMLOs were performed N = 1 for each biomarker. Images shown are representative of the populations. Individual scale bars provided.

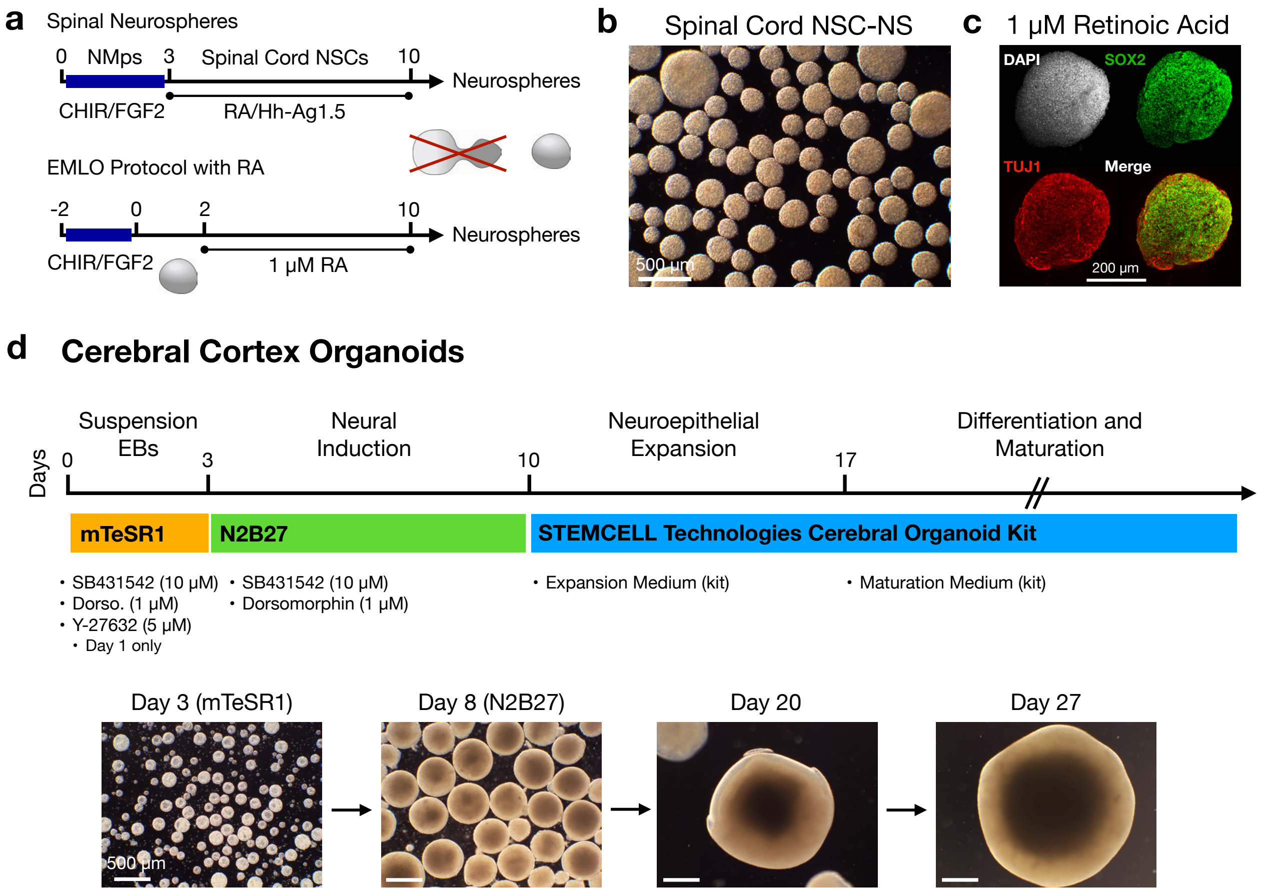

a
Spinal Neurospheres
0 NMps 3 Spinal Cord NSCs 10
Neurospheres
CHIR/FGF2
RA/Hh-Ag1.5
EMLO Protocol with RA
-2 0 2 10
Neurospheres
CHIR/FGF2
1 µM RA
b
Spinal Cord NSC-NS
500 µm
c
1 µM Retinoic Acid
DAPI
SOX2
TUJ1
Merge
200 µm
d
Cerebral Cortex Organoids
Suspension EBs
Neural Induction
Neuroepithelial Expansion
Differentiation and Maturation
Days
0 3 10 17
mTeSR1
N2B27
STEMCELL Technologies Cerebral Organoid Kit
• SB431542 (10 µM)
• Dorso. (1 µM)
• Y-27632 (5 µM)
• Day 1 only
• SB431542 (10 µM)
• Dorsomorphin (1 µM)
• Expansion Medium (kit)
• Maturation Medium (kit)
Day 3 (mTeSR1)
Day 8 (N2B27)
Day 20
Day 27
500 µm

Supplementary Fig. 3 Non-elongating stem cell models. a, Experimental overview of small molecule manipulation to prevent elongation. **b**, Phase contrast image of spinal cord NSC-derived neurospheres (NS). **c**, NMP aggregates coaxed into neurospheres by 1 μM retinoic acid (RA) added at day 2. **d**, Cerebral organoids do not elongate. Protocol adapted from Trujillo et al. (2019)[2], with the additional use of STEMCELL Technologies Cerebral Organoid kit. Each experiment in this figure was performed N = 2 separate times. Individual scale bars provided.

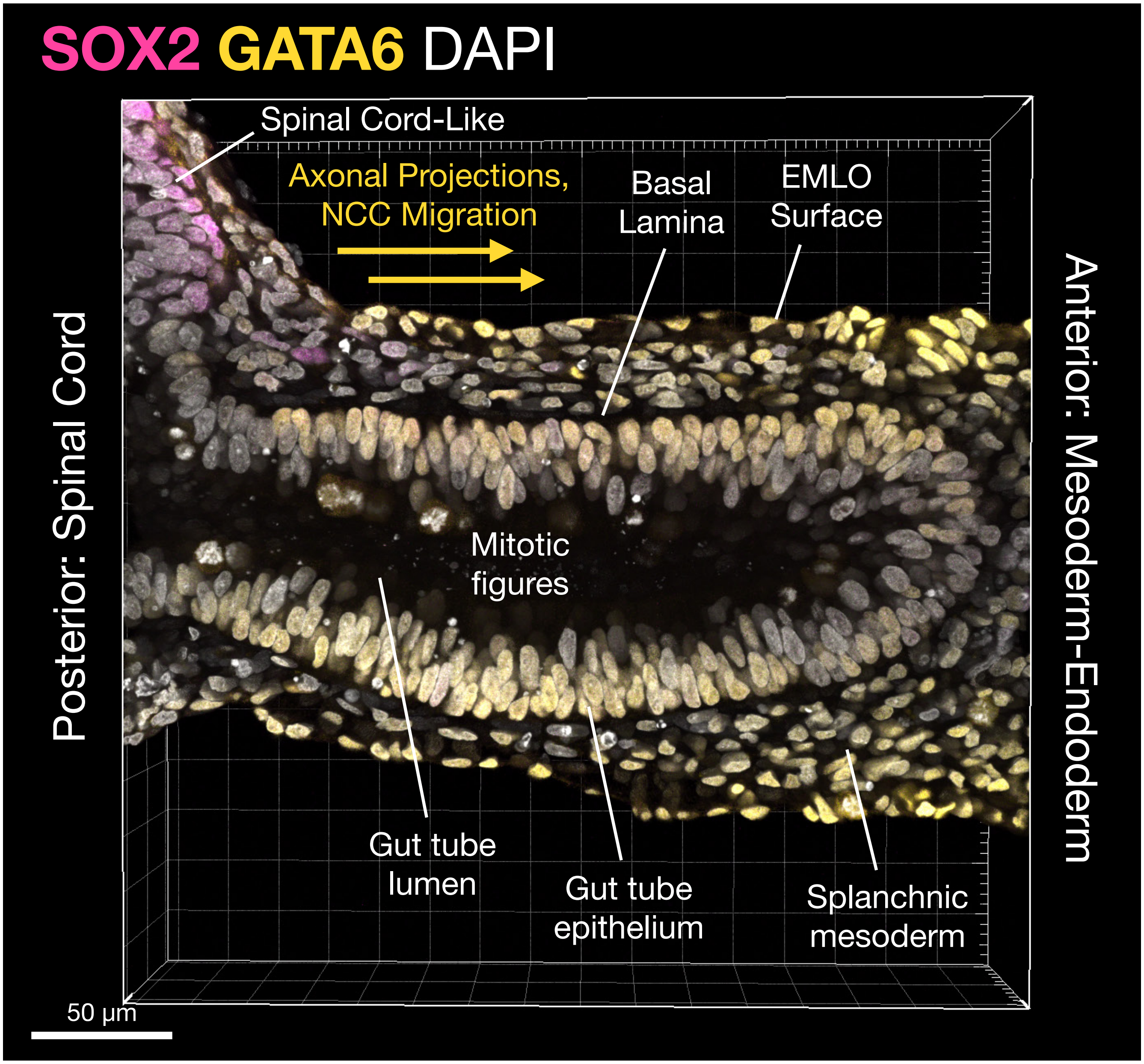

SOX2 GATA6 DAPI
Spinal Cord-Like
Axonal Projections, NCC Migration
Basal Lamina
EMLO Surface
Posterior: Spinal Cord
Anterior: Mesoderm-Endoderm
Mitotic figures
Gut tube lumen
Gut tube epithelium
Splanchnic mesoderm
50 μm

Supplementary Fig. 4 Annotated EMLO. Labeled EMLO depicting key structural and morphogenetic features. See also **Fig. 2d**. Scale bar is 50 μm.

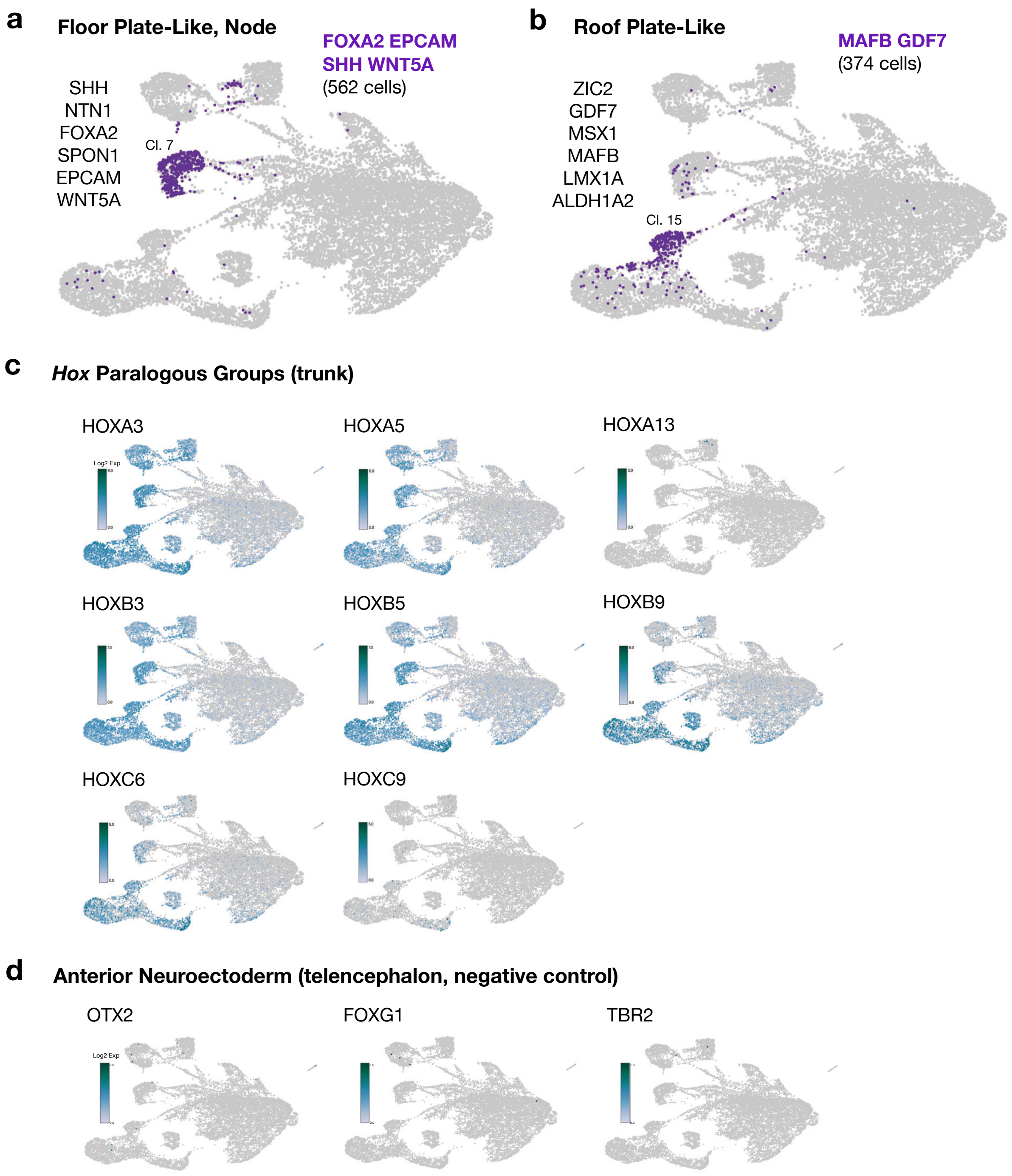
a
Floor Plate-Like, Node
FOXA2 EPCAM
SHH WNT5A
(562 cells)
SHH
NTN1
FOXA2
SPON1
EPCAM
WNT5A
Cl. 7
b
Roof Plate-Like
MAFB GDF7
(374 cells)
ZIC2
GDF7
MSX1
MAFB
LMX1A
ALDH1A2
Cl. 15
c
Hox Paralogous Groups (trunk)
HOXA3
HOXA5
HOXA13
Log2 Exp
HOXB3
HOXB5
HOXB9
HOXC6
HOXC9
d
Anterior Neuroectoderm (telencephalon, negative control)
OTX2
FOXG1
TBR2
Log2 Exp

Supplementary Fig. 5 Validation of posterior trunk versus anterior telencephalon CNS identity in day 16 EMLOs by scRNAseq. a, Annotation of cluster 7 as a floor plate- and node-like signaling center by combined expression of *FOXA2*, *EPCAM*, *SHH*, *WNT5A*. A list of associated genes is shown on the left. **b**, Annotation of cluster 15 as a roof plate-like signaling center by combined expression of *MAFB* and *GDF7*. A list of associated genes is shown on the left. **c**, *Hox* paralogous group analysis by scRNAseq indicates trunk identity. **d**, Absence of anterior neuroectodermal transcripts *OTX2*, *FOXG1* and *TBR2* supports trunk identity and has been previously observed[3,4].

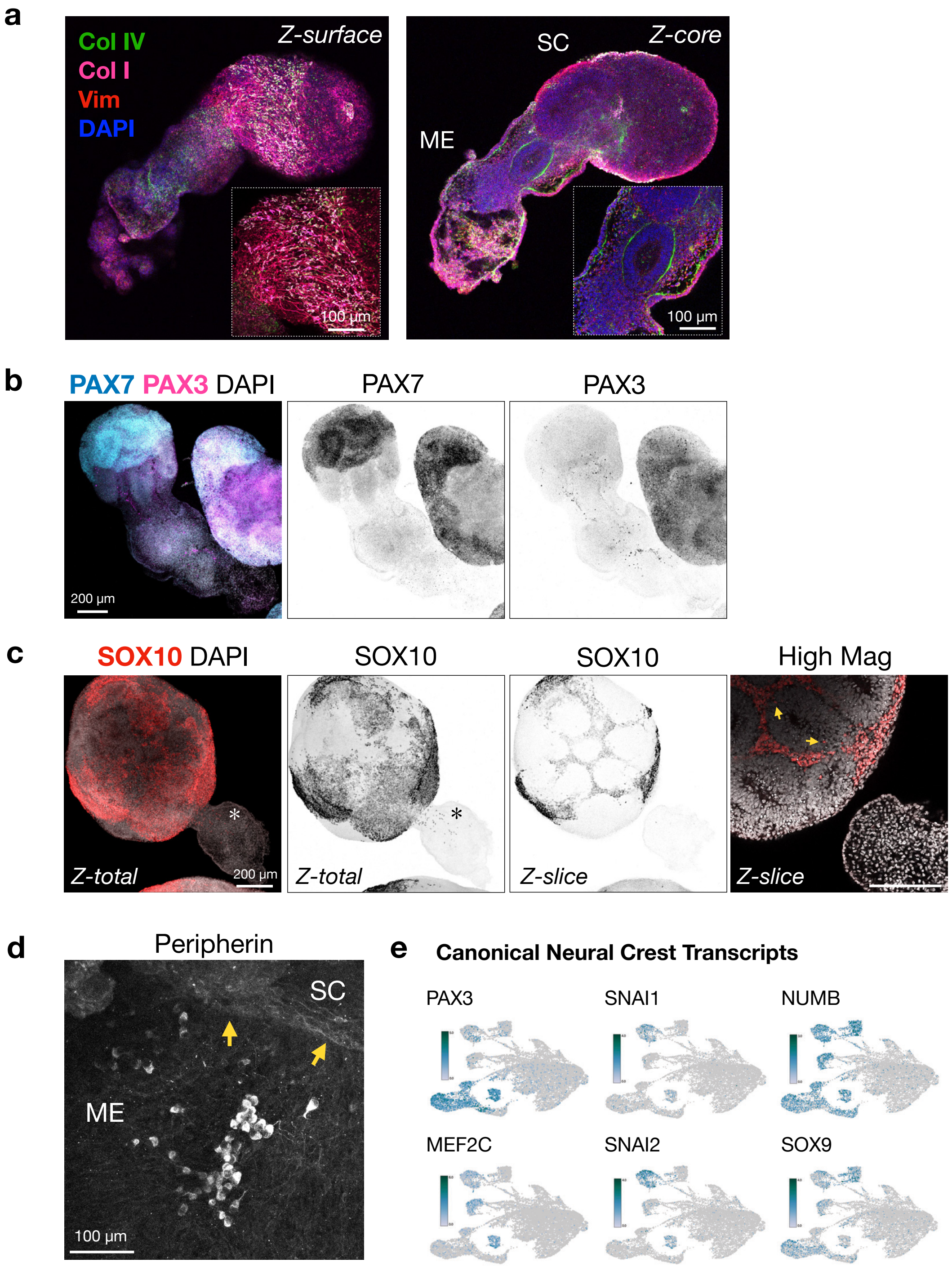
a
Col IV
Col I
Vim
DAPI
Z-surface
100 µm
SC
Z-core
ME
100 µm
b
PAX7 PAX3 DAPI
PAX7
PAX3
200 µm
c
SOX10 DAPI
SOX10
SOX10
High Mag
Z-total
200 µm
Z-total
Z-slice
Z-slice
d
Peripherin
SC
ME
100 µm
e
Canonical Neural Crest Transcripts
PAX3
SNAI1
NUMB
MEF2C
SNAI2
SOX9

Supplementary Fig. 6 Additional validation of NCCs and spinal cord identity in EMLOs. a, Left: EMLO exterior Z-slice depicts transition zone from SC to ME marked by Vimentin indicating active EMT. Inset is high magnification. Vimentin and ECM collagen biomarkers were stained in H3.3.1 EMLOs in N = 3/11 separate formation experiments. Right: EMLO interior Z-slice demonstrating basement membrane of gut tube marked by Collagen IV. Inset is high magnification. **b**, PAX7/PAX3 IF supports spinal cord identity of neural compartment (PAX7) and peripherally distributed PAX3+ cells in EMLOs. Non-elongated organoid shown to the right has uniform PAX3 distribution (internal control). PAX3 and PAX7 biomarkers were stained in H3.3.1 EMLOs in N = 2/11 repeat experiments. **c**, Maximally-projected Z-stack (Z-total) of SOX10 IF highlights NCC population of ME. Single Z-slices (right) shows interpenetration of SOX10+ cells between neuroectodermal rosettes in SC. Yellow arrows point to individual NCC nuclei within the rosette. NCC biomarkers were stained in H3.3.1 EMLOs in N = 8 of 11 formation experiments, and N = 3 formation experiments for the other representative lines **d**, Peripherin immunostain is observed in the ME at the SC-ME junction (see also **Fig. 8c** for further ME staining) (N = 2/11 repeat experiments). **e**, Canonical NCC transcripts by scRNAseq in day 16 H3.3.1 EMLOs. Individual scale bars provided.

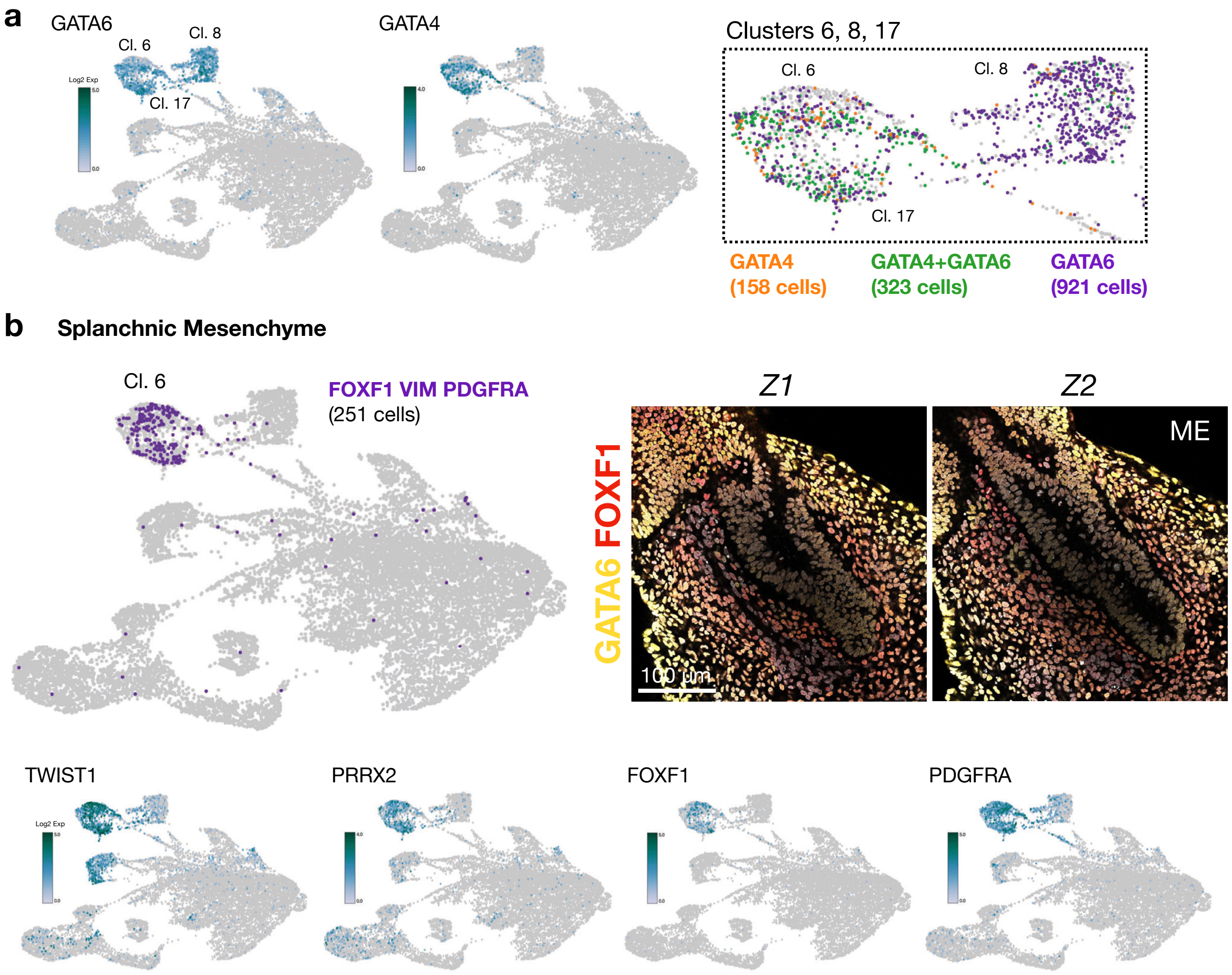
a
GATA6
Cl. 6
Cl. 8
Cl. 17
Log2 Exp
GATA4
Clusters 6, 8, 17
Cl. 6
Cl. 8
Cl. 17
GATA4 (158 cells)
GATA4+GATA6 (323 cells)
GATA6 (921 cells)
b
Splanchnic Mesenchyme
Cl. 6
FOXF1 VIM PDGFRA
(251 cells)
Z1
Z2
ME
GATA6 FOXF1
100 µm
TWIST1
PRRX2
FOXF1
PDGFRA
Log2 Exp

Supplementary Fig. 7 The EMLO gut tube is enveloped by splanchnic mesenchyme in ME. **a**, *GATA6* vs. *GATA4* expression in day 16 H3.3.1 EMLOs by scRNAseq. Clusters 6, 8, and 17 are expanded. **b**, Splanchnic mesenchyme was identified using scRNAseq by co-expression of *FOXF1*, *VIM*, and *PDGFRA* (251 cells)[5]. IF of *FOXF1* and *GATA6* provides further evidence as well as transcript expression of *TWIST1* and *PRRX2*. IF of the combined biomarkers *FOXF1* and *GATA6* was performed in N = 1 experiment and validated by scRNAseq (n = 15,576 H3.3.1 cells). Individual scale bars provided.

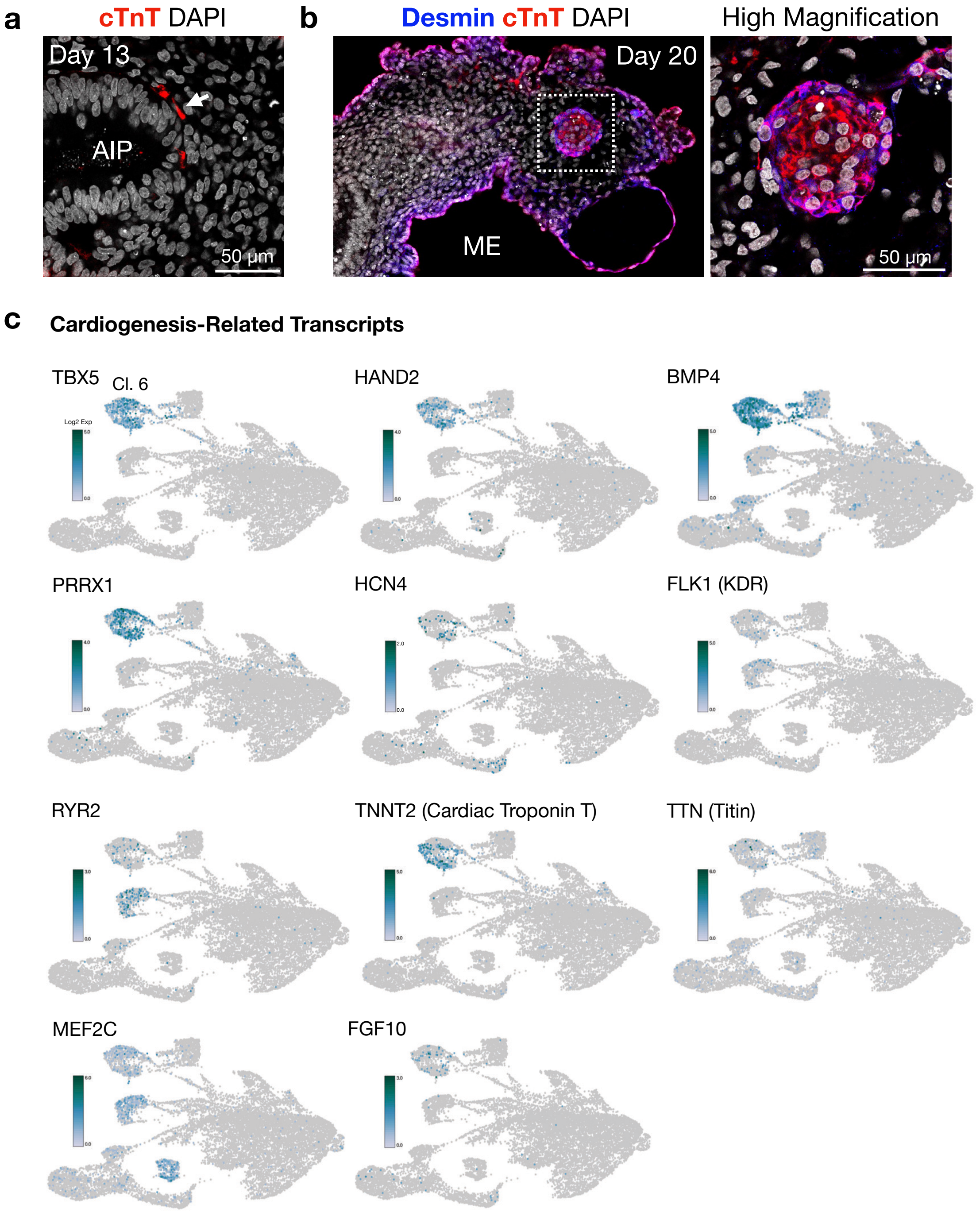
a
cTnT DAPI
Day 13
AIP
50 µm
b
Desmin cTnT DAPI
Day 20
ME
High Magnification
50 µm
c
Cardiogenesis-Related Transcripts
TBX5
Cl. 6
HAND2
BMP4
PRRX1
HCN4
FLK1 (KDR)
RYR2
TNNT2 (Cardiac Troponin T)
TTN (Titin)
MEF2C
FGF10

Supplementary Fig. 8 EMLOs permit cardiomyocyte differentiation. a, Cardiac Troponin-T (cTnT) adjacent to anterior intestinal portal-like region (day 13). **b**, Desmin/cTnT expression in ME towards base of gut tube. For each timepoint, cardiogenesis biomarker cTnT was stained for IF in N = 2/11 H3.3.1 formation experiments and additional biomarkers were validated by scRNAseq (**c**). Individual scale bars provided. **c**, Cardiogenesis-related transcripts by scRNAseq in day 16 H3.3.1 EMLOs.

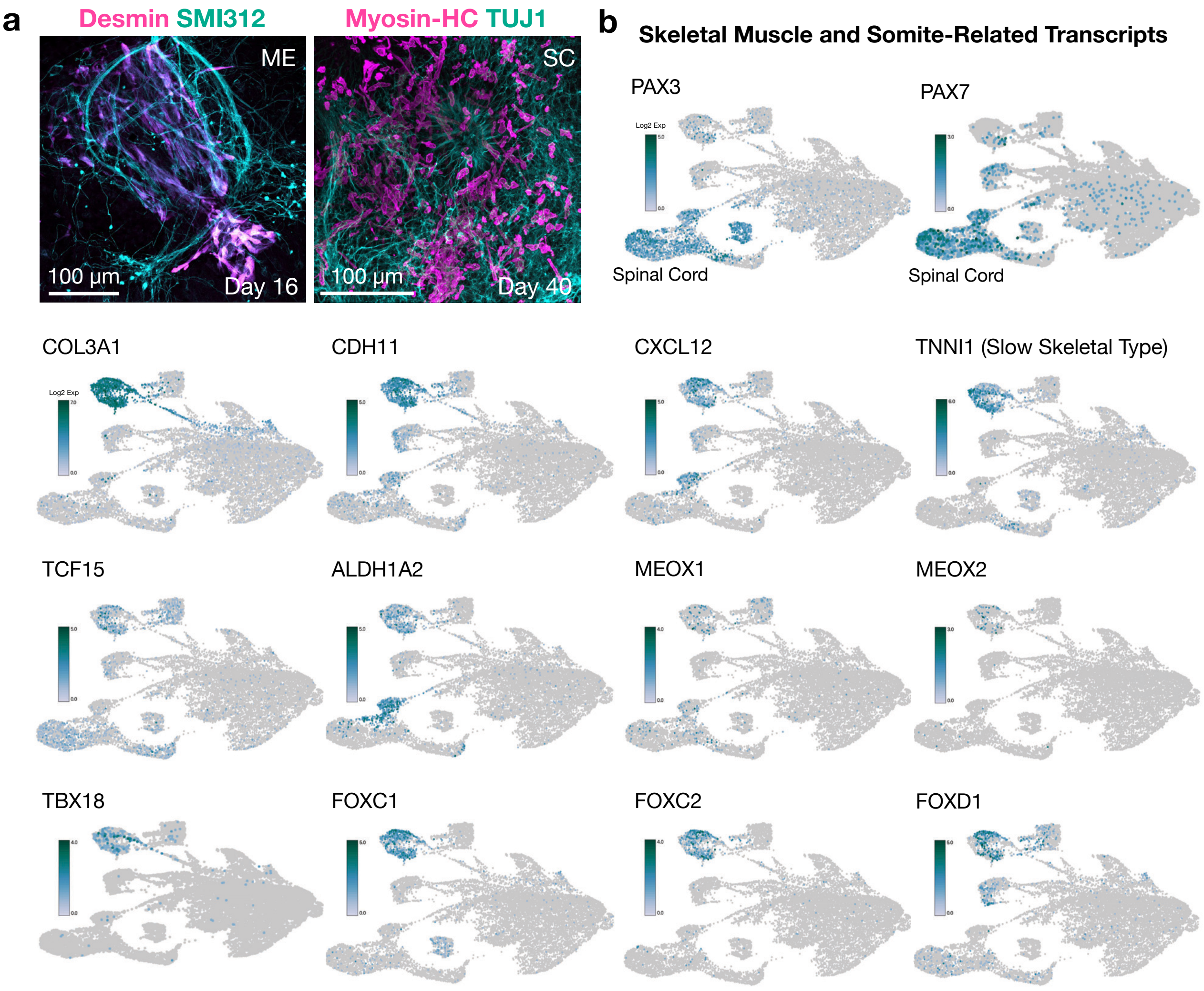
a
Desmin SMI312
Myosin-HC TUJ1
ME
SC
100 µm
Day 16
100 µm
Day 40
b
Skeletal Muscle and Somite-Related Transcripts
PAX3
PAX7
Log2 Exp
Spinal Cord
Spinal Cord
COL3A1
CDH11
CXCL12
TNNI1 (Slow Skeletal Type)
TCF15
ALDH1A2
MEOX1
MEOX2
TBX18
FOXC1
FOXC2
FOXD1

Supplementary Fig. 9 EMLOs permit skeletal muscle differentiation. a, Myogenic progenitors (Desmin) in an axon-rich (SMI312) region of ME in day 16 EMLO (left) and Myosin-HC expression in SC at day 40 (right). For each timepoint, skeletal myogenesis biomarkers were stained for IF in N = 2/11 H3.3.1 formation experiments. Biomarkers were validated by scRNAseq (**b**). Individual scale bars provided. **b**, Skeletal muscle and somite-related transcripts by scRNAseq in day 16 H3.3.1 EMLOs. At day 16, we did not detect evidence of the segmentation clock (*HES7*), suggesting that EMLO Matrigel embedding at earlier time points may be necessary to induce segmentation mechanisms.

SUPPLEMENTARY TABLES

Supplementary Table 1. Ethnically-Diverse hiPSC Lines

Line	Passage number	Pluripotency	Teratoma	Multi-lineage differentiation	hiPSC bulk RNA-Seq	ChIP-Seq	EMLO formation
**F3.5.2**	15-25	IF, RT-PCR	Yes	Tomov et al. 2016[6]	Tomov et al. 2016[6]	Tomov et al. 2016[6]	Yes
F3.2.2	7-8	IF, RT-PCR	Not tested	This study	Not tested	Not tested	Yes
F3.3.1	10-11	IF, RT-PCR	Not tested	This study	Not tested	Not tested	Yes
**H3.3.1**	7-11	IF, RT-PCR	Yes	Tomov et al. 2016[6]	Tomov et al. 2016[6]	Tomov et al. 2016[6]	Yes
H3.1.1	8-11	IF, RT-PCR	Yes	Tomov et al. 2016[6]	Tomov et al. 2016[6]	Tomov et al. 2016[6]	Yes
H3.4.1	6-9	IF, RT-PCR	Not tested	This study	Not tested	Not tested	Yes
**A2.1.1**	11-14	IF, RT-PCR	Yes	Tomov et al. 2016[6]	Tomov et al. 2016[6]	Tomov et al. 2016[6]	Yes
A2.2.1	6-9	IF, RT-PCR	Yes	This study	Not tested	Not tested	Yes
A2.2.2	10-17	IF, RT-PCR	Yes	Tomov et al. 2016[6]	Tomov et al. 2016[6]	Tomov et al. 2016[6]	Yes

Supplementary Table 2. Optimizing EMLO Conditions to Promote or Inhibit Elongation

Condition	Promote	Inhibit	Comment	Developmental Rationale
Low cell number (300-400)			Starting aggregate	Gastrula-like; optimal length scale for internal signal induction, exposure to external factors
High cell number (9-10,000)			Starting aggregate	Tissue-like, differential exposure to external factors
CHIR/FGF pretreatment as single cells			Advantage: homogenous NMPs	Homogenous induction, initial cell-cell adhesion lost
CHIR/FGF pretreatment as 2D colonies			Advantage: mesendoderm, early NMP	Initial cell-cell adhesion retained
CHIR/FGF longer induction			3 days	NMPs more induced
CHIR/FGF shorter induction			2 days	NMPs less induced
Direct to shaking cultures (day 0)			Advantage: EMLOs	Optimized to ensure appropriate cell number
Direct to 96-well plate static			Advantage: neuromuscular trunk organoids	Aggregation of larger cell number (4.5-9,000) for organoids
Higher culture density			6 well plate after day 4	Density effects on paracrine signaling and nutrient availability
Lower culture density			100 mm dish after day 4	Density effects on paracrine signaling and nutrient availability
Fast shaking			90 rpm	Mechanical forces promote spheres
Slow shaking			70 rpm	Mechanical forces permissive to elongation

Early high-dose retinoic acid			Spinal cord neurospheres	Premature commitment to neuroectoderm
Aggregate formation with spinal cord NSCs			Spinal cord neurospheres	Formation from posterior neuroectoderm (no ME, no NMP)
Aggregate formation with anterior NSCs			Cerebral organoids	Formation from anterior neuroectoderm (telencephalic, no ME, no NMP)
Dual SMAD Inhibition			Day 2 early addition	Mesoderm, endoderm contributions to elongation reduced (TGF-β, BMP signal inhibition)

Supplementary Table 3. EMLO Qualitative Biomarker Summary and Signficance*

Target	Neural (SC)	Mesoderm-Endoderm (ME, total)	Gut Tube Epithelium	Ganglion (Yes/No)	Significance	Distribution/ Comments
SOX2	++++	++	+	No	Pluripotency, neural stem cells, gastrointestinal	Highly expressed in SC, basal levels in gut tube
CDH2 (N-Cadherin)	++++	+	-	Yes	Neural cell adhesion	SC rosettes and base of gut tube (anterior)
TUJ1 (TUBB3)	++++	+++	+	Yes	Post-mitotic neurons	SC, concentrated in +EMT transition zone, gut tube envelopment, peripheral ganglia, other ME projections
GAP-43	+	++++	-	Yes	Axon pathfinding	Axons enriched in ME
SMI312	++	++	-	Yes	Axons	SC, ME
LHX9	++	-	-	No	Dorsal spinal cord (dI1)	SC
LBX1	++	-	-	No	Dorsal-intermediate spinal cord (dI4, dI6)	SC
PAX2	++++	-	-	No	Dorsal-intermediate spinal cord (dI4, dI6, V0, V1)	SC
CHX10	+	-	-	No	Ventral spinal cord interneuron (V2a)	SC
NKX6-1	+	-	-	No	Ventral spinal cord motor neuron (MN)	SC
OPRM1	+++	++	+	No	Mu opioid receptor	SC apical aspect of rosettes, neurons at rosette base, gut tube lumen
GATA6	-	++++	++	No	Heart fields, pancreas, diaphragm, liver, vasculature	ME gut tube plus mesenchyme
GATA4	-	++++	-	No	Endoderm specification, splanchnic mesoderm, valves, heart fields, diaphragm	ME mesenchyme only

FOXF1	+++	+++	+	No	Splanchnic mesoderm, mesenchyme	SC, ME basal levels in gut tube, highly expressed in mesenchyme
ISL1	+++	++	+	Yes	MNs, dorsal INs, cardiac NCC, NCC-derived neurons	SC, gut tube, enriched at AIP and buds, peripheral ganglia
TFAP2α	++++	++	-	Yes	GABAergic neuronal progenitors, NCC, PNS	SC at base of rosettes, ME distribution along gut tube, peripheral ganglia, ME non-ganglia
SOX10	++++	++	-	No	NCC, PNS	SC at base of rosettes, concentrated EMT transition zone, ME along gut tube
PAX7	+++	-	-	No	Dorsal spinal cord, myogenic progenitors	SC before EMT transition zone
PAX3	+	++	-	No	Migratory NCC, myogenic progenitors	ME along gut tube; uniform in spheroids/ovoids
Vimentin	++	++	-	No	EMT, myogenic progenitors	EMT transition zone, ME
Peripherin	-	+	-	Yes	PNS (vs. CNS)	ME not SC
Type IV Collagen	+	+	-	No	Basement membrane, EMT	EMT transition zone, basal lamina, gut tube, surface layer ME
FOXA2/ HNF-3β	+	+++	+++	No	Definitive endoderm	Spans SC to ME in gut tube
CDH1 (E-Cadherin)	++	++	+++	No	Epithelial	Gut tube cell-cell adhesion, surface layer ME
Desmin	+	++	-	No	Myogenic progenitors	SC, ME
Myosin Heavy Chain	+	+	-	No	Muscle cells	SC, ME
Troponin-T	-	+	-	No	Cardiomyocytes	Base of anterior intestinal portal-like region

*Qualitative scale (++++, +++, ++, +, -) by day 22

SUPPLEMENTARY REFERENCES

1. Faustino Martins, J.M. et al. Self-organizing 3D human trunk neuromuscular organoids. *Cell Stem Cell* **26,** 172-186 (2020).

2. Trujillo, C.A. et al. Complex oscillatory waves emerging from cortical organoids model early human brain network development. *Cell Stem Cell* **25,** 558-569 (2019).

3. van den Brink, S.C. et al. Symmetry breaking, germ layer specification and axial organization in aggregates of mouse embryonic stem cells. *Development* **141,** 4231-4242 (2014).

4. Moris, N. et al. An in vitro model of early anteroposterior organization during human development. *Nature* **582,** 410-415 (2020).

5. Han, L. et al. Single cell transcriptomics identifies a signaling network coordinating endoderm and mesoderm diversification during foregut organogenesis. *Nat. Commun.* **11,** 4158 (2020).

6. Tomov, M.L. et al. Distinct and shared determinants of cardiomyocyte contractility in multi-lineage competent ethnically diverse iPSCs. *Sci. Rep.* **6,** 37637 (2016).
