# Supplementary material for: Co-development of central and peripheral neurons with trunk mesendoderm in human elongating multi-lineage organized gastruloids
Source: Nat Commun. 2021 May 21;12:3020. doi: 10.1038/s41467-021-23294-7 (PMC8140076; doi:10.1038/s41467-021-23294-7)
Supplement: Supplementary file 9 — Reporting Summary [file 41467_2021_23294_MOESM9_ESM.pdf]

nature research

Corresponding author(s): Janet Paluh

Last updated by author(s): Apr 6, 2021

Reporting Summary

Nature Research wishes to improve the reproducibility of the work that we publish. This form provides structure for consistency and transparency in reporting. For further information on Nature Research policies, see our Editorial Policies and the Editorial Policy Checklist.

Statistics

For all statistical analyses, confirm that the following items are present in the figure legend, table legend, main text, or Methods section.

n/a	Confirmed	
☐	☒	The exact sample size (*n*) for each experimental group/condition, given as a discrete number and unit of measurement
☐	☒	A statement on whether measurements were taken from distinct samples or whether the same sample was measured repeatedly
☐	☒	The statistical test(s) used AND whether they are one- or two-sided<br>*Only common tests should be described solely by name; describe more complex techniques in the Methods section.*
☒	☐	A description of all covariates tested
☒	☐	A description of any assumptions or corrections, such as tests of normality and adjustment for multiple comparisons
☐	☒	A full description of the statistical parameters including central tendency (e.g. means) or other basic estimates (e.g. regression coefficient) AND variation (e.g. standard deviation) or associated estimates of uncertainty (e.g. confidence intervals)
☐	☒	For null hypothesis testing, the test statistic (e.g. *F*, *t*, *r*) with confidence intervals, effect sizes, degrees of freedom and *P* value noted<br>*Give P values as exact values whenever suitable.*
☒	☐	For Bayesian analysis, information on the choice of priors and Markov chain Monte Carlo settings
☒	☐	For hierarchical and complex designs, identification of the appropriate level for tests and full reporting of outcomes
☒	☐	Estimates of effect sizes (e.g. Cohen's *d*, Pearson's *r*), indicating how they were calculated

Our web collection on statistics for biologists contains articles on many of the points above.

Software and code

Policy information about availability of computer code

Data collection	We did not generate new code for this study, and provide the software used in the Key Resources table in Supplementary Information<br><br>cellSens Olympus (V2.3, 64 bit) https://www.olympus-lifescience.com/en/software/cellsens/<br>Leica Application Suite (v2.7.3.9723) https://www.leica-microsystems.com/products/microscope-software/p/leica-application-suite/<br>10x Genomics Cell Ranger (V5.0.0) https://support.10xgenomics.com/<br>Zeiss AxiovsionRel (v4.8.1) https://www.micro-shop.zeiss.com/en/us/system/software/software+axiovision/axiovision +program/410130-0909-000
Data analysis	We did not generate new code for this study, and provide the software used in the Key Resources table in Supplementary Information<br>GraphPad Prism 9 (V9.0.2) GraphPad https://www.graphpad.com/scientific-software/prism/<br>Microsoft Excel (V16.16.27) https://www.microsoft.com/en-us/microsoft-365/excel<br>Imaris BitPlane (V7.6.0, 64 bit) https://imaris.oxinst.com/<br>Loupe Browser 10x Genomics (V5.0.0) https://support.10xgenomics.com/<br>Fiji Schindelin et al., 2012 https://imagej.net/Fiji<br>Adobe Illustrator (V25.2.1) https://www.adobe.com/products/illustrator/<br>Apple Keynote (V10.3.8) https://www.apple.com/keynote/

For manuscripts utilizing custom algorithms or software that are central to the research but not yet described in published literature, software must be made available to editors and reviewers. We strongly encourage code deposition in a community repository (e.g. GitHub). See the Nature Research guidelines for submitting code & software for further information.

Data

Policy information about availability of data

All manuscripts must include a data availability statement. This statement should provide the following information, where applicable:

- Accession codes, unique identifiers, or web links for publicly available datasets
- A list of figures that have associated raw data
- A description of any restrictions on data availability

The authors declare that all data supporting the findings of this study are available within the article and its Supplementary Information files or from the corresponding author upon reasonable request. Source data is included with this manuscript (Supplementary Data 2). We have deposited the single cell sequencing data to Gene Expression Omnibus (GEO) under the ID code: GSE166603.

Field-specific reporting

Please select the one below that is the best fit for your research. If you are not sure, read the appropriate sections before making your selection.

[x] Life sciences [ ] Behavioural & social sciences [ ] Ecological, evolutionary & environmental sciences

For a reference copy of the document with all sections, see nature.com/documents/nr-reporting-summary-flat.pdf

Life sciences study design

All studies must disclose on these points even when the disclosure is negative.

Sample size	We did not perform power analysis to determine sample size since animals and human subjects were not used. Statistics for counting cells used sufficient numbers for analysis and source data is provided with this manuscript. Cell counts totaled >6,000 per condition to include sample sizes that are comparable other know methods with high statistical power such as single cell RNA sequencing. For our single cell sequencing data set, the day 16 EMLO sample totaled 15,576 cells. We used comparable sample sizes to other gastruloid studies (van den Brink et al., 2014; Moris et al., 2020; Rossi et al., 2021).
Data exclusions	Log2Exp > 0 or =0 were used for Boolean filters of scRNAseq data in Loupe software
Replication	Replicate experiments performed were done so as new EMLO formation experiments or their application. All attempts at repeats were successful. H3.3.1 EMLOs were formed in N = 11 separate biological repeat experiments over the course of this study with similar results. Phase and IF data were acquired each time. EMLOs from the other F3.5.2 and A2.1.1 representative lines were separately formed in N = 5 biological repeat experiments. All other lines were formed in N = 3 repeat experiments. Images are representative of the EMLO populations across repeated experiments. Details for individual experiments and antibody staining is provided in the corresponding figure legends.
Randomization	This study was not designed to be randomized since this technique was not applicable to the type of study. No animals or human subjects were involved. Randomization was not necessary to preserve the legitimacy and strength of the findings.
Blinding	Analysis was not blinded. Dr. Paluh and Olmsted performed the analyses. Cell numbers were measured according to consistent methods. Other measured EMLO parameters included cell size parameters, morphology, and distribution that were measured in Fiji ImageJ by the authors and so were not blinded.

Reporting for specific materials, systems and methods

We require information from authors about some types of materials, experimental systems and methods used in many studies. Here, indicate whether each material, system or method listed is relevant to your study. If you are not sure if a list item applies to your research, read the appropriate section before selecting a response.

Materials & experimental systems

n/a	Involved in the study
[ ]	[x] Antibodies
[ ]	[x] Eukaryotic cell lines
[x]	[ ] Palaeontology and archaeology
[x]	[ ] Animals and other organisms
[x]	[ ] Human research participants
[x]	[ ] Clinical data
[x]	[ ] Dual use research of concern

Methods

n/a	Involved in the study
[x]	[ ] ChIP-seq
[x]	[ ] Flow cytometry
[x]	[ ] MRI-based neuroimaging

Antibodies

Antibodies used	A key resources table is provided in the Supplementary Information file. All antibodies are listed with catalog numbers and RRIDs if

available.

Rat Anti-SSEA3 (AF488 conjugate) STEMCELL Technologies (Cat.No.: 60061AD; RRID: AB_1118554); Clone: MC-631
Mouse anti-SSEA4 STEMCELL Technologies (Cat.No.: 60062; RRID: AB_2721031); Clone MC-813-70
Mouse anti-SOX2 R&D Systems (Cat.No.: MAB2018; RRID: AB_358009); Clone #245610
Goat anti-SOX2 R&D Systems (Cat.No.: AF2018; RRID: AB_355110)
Goat anti-Brachyury R&D Systems Cat.No.: AF2085; RRID: AB_2200235
Goat anti-GATA4 R&D Systems Cat.No.: AF2606; RRID: AB_2232177
Goat anti-GATA6 R&D Systems Cat.No.: AF1700; RRID: AB_2108901
Mouse anti-CDX2 DSHB (Cat.No.: PCRP-CDX2-1A3; RRID: CVCL_KD04); Clone: 1A3
Rabbit anti-ISL1 Sigma-Aldrich Cat.No.: HPA057416; RRID: AB_2683431
Mouse anti-TFAP2a DSHB (Cat.No.: 3B5; RRID: AB_667767); Clone: 3B5
Goat anti-SOX10 R&D Systems Cat.No.: AF2864; RRID: AB_442208
Mouse anti-Collagen IV a1 R&D Systems (Cat.No.: MAB6308); Clone: #577238
Rabbit anti-Collagen I a1 Novus Biologicals Cat.No.: NBP1-30054; RRID: AB_1968486
Goat anti-Vimentin R&D Systems Cat.No.: AF2105; RRID: AB_355153
Goat anti-CDH1/E-cadherin R&D Systems Cat. No.: AF648; RRID: AB_355504
Goat anti-FOXA2 R&D Systems Cat.No.: AF2400; RRID: AB_2294104
Rabbit anti-FOXF1 Abcam Cat.No.: ab168383
Goat anti-SOX17 R&D Systems Cat.No.: AF1924; RRID: AB_355060
Rabbit anti-b-tubulin III/TUJ1 (TUBB3) BioLegend Cat.No.: 802001; RRID: AB_2564645
Mouse anti-b-tubulin III/TUJ1 (TUBB3) Abcam (Cat.No.: ab7751; RRID: AB_306045); Clone: TU-20
Mouse SMI312 cocktail BioLegend Cat.No.: 837904; RRID: AB_2566782
Mouse anti-GAP-43 Encor (Cat.No.: MCA-5E8; RRID: AB_2572287)
Mouse anti-Peripherin Santa Cruz Biotechnologies (Cat.No.: sc-377093); Clone: A-3
Rabbit anti-CDH2/N-cadherin Cell Signaling Technologies Cat.No.: 13116; RRID: AB_2687616
Mouse anti-ZO1 Invitrogen (Cat.No.: 33-9100; RRID: AB_2533147); Clone: ZO1-1A12
Rabbit anti-Mu Opioid Receptor (OPRM1) Abcam Cat.No.: ab10275; RRID: AB_2156356
Rabbit anti-GAD65&67 Abcam Cat.No.: ab11070; RRID: AB_297722
Rabbit anti-TLX3 Abcam Cat.No.: ab184011
Rabbit anti-LBX1 Invitrogen Cat.No.: PA564884; RRID: AB_2662958
Rabbit anti-PAX2 Invitrogen Cat.No.: 716000; RRID: AB_2533990
Goat anti-PAX3 R&D Systems Cat.No.: AF2457; RRID: AB_416599
Mouse anti-PAX7 R&D Systems (Cat.No.: MAB1675; RRID: AB_2159833); Clone: #PAX7
Rabbit anti-LHX9 Abcam Cat.No.: ab224357
Mouse anti-CHX10 Santa Cruz Biotechnologies (Cat.No.: sc-374151); Clone: D-11
Mouse anti-NKX6.1 DSHB (Cat.No.: F55A12; RRID: AB_532379); Clone: F55A12
Rabbit anti-BRN3A EMD Millipore Cat.No.: AB5945; RRID: 92154
Goat anti-BMP2/4 R&D Systems Cat.No.: AF355
Goat anti-Desmin R&D Systems Cat.No.: AF3844; RRID: AB_2092419
Mouse anti-Myosin Heavy Chain R&D Systems (Cat.No.: MAB4470; RRID: AB_1293549); Clone: #MF20
Mouse anti-Troponin T (cardiac) R&D Systems (Cat.No.: MAB1874); Clone: #200805

Secondary antibodies:
Goat anti-Mouse Cy5 Life Technologies (cat. no.: A10524; lot: 2155288)
Goat anti-Rabbit Cy5 Life Technologies (cat. no.: A10523; lot: 2156245)
Donkey anti-Goat AlexaFluor 594 Life Technologies (cat.no.: A32758; lot: VE300592B)
Donkey anti-Rabbit AlexaFluor 488 Life Technologies (cat. no.: A21206; lot: 2156521)

Validation

Rat Anti-SSEA3 (AF488 conjugate) STEMCELL Technologies Cat.No.: 60061AD; validated in this study and on the manufacturer's website (3 fluorescence data examples), no other references available
Mouse anti-SSEA4 STEMCELL Technologies Cat.No.: 60062; RRID: AB_2721031; 6 publications listed on the manufacturer's website
Mouse anti-SOX2 R&D Systems Cat.No.: MAB2018; RRID: AB_358009; 121 publications listed on the manufacturer's website
Goat anti-SOX2 R&D Systems Cat.No.: AF2018; RRID: AB_355110; 93 publications listed on the manufacturer's website
Goat anti-Brachyury R&D Systems Cat.No.: AF2085; RRID: AB_2200235; 72 publications listed on the manufacturer's website, 5 different data examples (3 ICC, 1 WB, 1 ChiP)
Goat anti-GATA4 R&D Systems Cat.No.: AF2606; RRID: AB_2232177; 18 publications manufacturer's website, 4 different data examples (2 ICC, 1 WB, 1 ChiP)
Goat anti-GATA6 R&D Systems Cat.No.: AF1700; RRID: AB_2108901; 32 publications listed on the manufacturer's website, 4 different data examples (2 ICC, 1 WB, 1 ChiP)
Mouse anti-CDX2 DSHB Cat.No.: PCRP-CDX2-1A3; RRID: CVCL_KD04; 1 publication listed on the manufacturer's website. This manufacturer uses highly characterized mouse monoclonal antibodies from hybridomas
Rabbit anti-ISL1 Sigma-Aldrich Cat.No.: HPA057416; RRID: AB_2683431; validated in this study and on the manufacturer's website, 2 different data examples (1 IHC, 1 ICC)
Mouse anti-TFAP2a DSHB Cat.No.: 3B5; RRID: AB_667767; 50 publications provided on the manufacturer's website. This manufacturer uses highly characterized mouse monoclonal antibodies from hybridomas
Goat anti-SOX10 R&D Systems Cat.No.: AF2864; RRID: AB_442208; 27 publications provided on the manufacturer's website, 1 data example (1 ICC)

Mouse anti-Collagen IV a1 R&D Systems Cat.No.: MAB6308; validated in this study and on the manufacturer's website, 2 different data examples (2 ICC). This antibody was designed to react specifically against human epitopes and was tested for cross-reactivity by ELISA according to the manufacturer's website.
Rabbit anti-Collagen I a1 Novus Biologicals Cat.No.: NBP1-30054; RRID: AB_1968486; 34 publications listed on the manufacturer's website
Goat anti-Vimentin R&D Systems Cat.No.: AF2105; RRID: AB_355153; 20 publications listed on the manufacturer's website, 6 different data examples (2 ICC, 2 KO validation, 2 IHC)
Goat anti-CDH1/E-cadherin R&D Systems Cat. No.: AF648; RRID: AB_355504; 21 publications listed on the manufacturer's website, 7 different data examples (2 WB, 1 FACS, 2 ICC, 1 IHC, 1 RNAscope)
Goat anti-FOXA2 R&D Systems Cat.No.: AF2400; RRID: AB_2294104; 38 publications listed on the manufacturer's website, 4 different data examples (1 WB, 1 ChiP, 1 ICC, 1 IHC)
Rabbit anti-FOXF1 Abcam Cat.No.: ab168383; 3 publications listed on the manufacturer's website, 3 different data examples (1 WB, 1 FACS, 1 ICC). The anti-FOXF1 antibody was validated on human HeLa cells for ICC/IF according to the manufacturer's website.
Goat anti-SOX17 R&D Systems Cat.No.: AF1924; RRID: AB_355060; 163 publications listed on the manufacturer's website, 6 different data examples (3 WB, 1 ChIP, 2 ICC)
Rabbit anti-b-tubulin III/TUJ1 (TUBB3) BioLegend Cat.No.: 802001; RRID: AB_2564645; 70 publications listed on the manufacturer's website, 4 different data examples (3 IHC, 1 WB)
Mouse anti-b-tubulin III/TUJ1 (TUBB3) Abcam Cat.No.: ab7751; RRID: AB_306045; 119 publications listed on the manufacturer's website
Mouse SMI312 cocktail BioLegend Cat.No.: 837904; RRID: AB_2566782; 38 publications listed on the manufacturer's website, 4 different data examples (3 IHC, 1 WB)
Mouse anti-GAP-43 Encor Cat.No.: MCA-5E8; RRID: AB_2572287; 6 publications listed on the manufacturer's website, 3 different data examples (2 ICC, 1 WB)
Mouse anti-Peripherin Santa Cruz Biotechnologies Cat.No.: sc-377093; 3 publications listed on the manufacturer's website, 12 different data examples (1 ICC, 1 IHC, 10 WB). Peripherin reacts with the human epitode and was validated in methanol-fixed human HeLa cell's on the manufacturer's website.
Rabbit anti-CDH2/N-cadherin Cell Signaling Technologies Cat.No.: 13116; RRID: AB_2687616; 428 publications listed on the manufacturer's website
Mouse anti-ZO1 Invitrogen Cat.No.: 33-9100; RRID: AB_2533147; >500 publications listed on the manufacturer's website
Rabbit anti-Mu Opioid Receptor (OPRM1) Abcam Cat.No.: ab10275; RRID: AB_2156356; 38 publications listed on the manufacturer's website, 3 different data examples (3 ICC)
Rabbit anti-GAD65&67 Abcam Cat.No.: ab11070; RRID: AB_297722; 42 publications listed on the manufacturer's website, 1 different data examples (1 WB)
Rabbit anti-TLX3 Abcam Cat.No.: ab184011; 1 publication listed on the manufacturer's website, 3 different data examples (1 WB, 2 IHC). The manufacturer declares that this antibody reacts with the human epitope of TLX3 and is suitable for staining fixed cells. The publication Wang et al. 2019 Int. J. Biol. Sci. (PMID: 31360112) uses this reagent.
Rabbit anti-LBX1 Invitrogen Cat.No.: PA564884; RRID: AB_2662958; validated in this study and 1 data example listed on manufacturer's website (1 ICC)
Rabbit anti-PAX2 Invitrogen Cat.No.: 716000; RRID: AB_2533990; 100 publications listed on the manufacturer's website, 9 different data examples
Goat anti-PAX3 R&D Systems Cat.No.: AF2457; RRID: AB_416599; 2 publications listed on the manufacturer's website
Mouse anti-PAX7 R&D Systems Cat.No.: MAB1675; RRID: AB_2159833; 26 publications listed on the manufacturer's website, 2 different data examples (2 ICC)
Rabbit anti-LHX9 Abcam Cat.No.: ab224357; 1 publication listen on the manufacturer's website, 3 different data examples (1 WB, 1 ICC, 1 IHC). This antibody was validated in human BJ cells by IF, prepared with PFA fixation, Triton X-100 permeabilization on the manufacturer's website.
Mouse anti-CHX10 Santa Cruz Biotechnologies Cat.No.: sc-374151; 7 publications listed on the manufacturer's website, 3 different data examples (2 WB, 1 IHC). This antibody is declared to react with the human epitope by IF according to the manufacturer's website.
Mouse anti-NKX6.1 DSHB Cat.No.: F55A12; RRID: AB_532379; 7 publications listed on the manufacturer's website
Rabbit anti-BRN3A EMD Millipore Cat.No.: AB5945; RRID: 92154; 6 publications listed on the manufacturer's website
Goat anti-BMP2/4 R&D Systems Cat.No.: AF355; 2 publications listed on the manufacturer's website. On this site, the antibody is declared to react specifically against the human epitope and was validated by immersion fixed BMP-2 transfected human cells.
Goat anti-Desmin R&D Systems Cat.No.: AF3844; RRID: AB_2092419; 13 publications listed on the manufacturer's website, 2 different data examples (1 ICC, 1 IHC)
Mouse anti-Myosin Heavy Chain R&D Systems Cat.No.: MAB4470; RRID: AB_1293549; 50 publications listed on the manufacturer's website, 3 different data examples (1 ICC, 2 IHC)
Mouse anti-Troponin T (cardiac) R&D Systems Cat.No.: MAB1874; 5 publications listed on the manufacturer's website, 2 different data examples (1 ICC, 1 IHC). According to the manufacturer's site, the antibody is specifically reactive to the human epitope and was validated by IF on human ESCs differentiated to cardiomyocytes and by IHC on human heart tissue.

Eukaryotic cell lines

Policy information about cell lines

Cell line source(s)	Donor fibroblasts (Coriell): F3 (cat. no. GM22268), H3 (GM22186), A2 (cat. no. AG08498)<br><br>Clonal iPS lines: F3.5.2, F3.2.2, F3.3.1; H3.3.1, H3.1.1, H3.4.1; A2.1.1, A2.2.1, A2.2.2<br>Chang et al., 2015 (PMID: 26482195)
Authentication	We derived and expanded the ED-iPSC lines from Coriell deidentified human fibroblast samples from consenting donors. ED-hiPSC lines were reprogrammed by the Cibelli and Paluh laboratories from these donor fibroblasts and comprehensively

	characterized by the follows methods (Supplementary Table 1):<br><br>Pluripotency (immunofluorescence, RT-PCR), G-band karyotype, teratoma formation, multi-lineage differentiation, bulk RNA-Seq, ChIP-Seq, EMLO formation
Mycoplasma contamination	Cell lines tested negative for mycoplasma contamination
Commonly misidentified lines<br>(See ICLAC register)	N/A - iPS lines derived from the Coriell fibroblasts of deidentified consenting donors are in the process of being deposited with WiCell (Madison, WI)
