# Supplementary material for: Co-development of central and peripheral neurons with trunk mesendoderm in human elongating multi-lineage organized gastruloids
Source: Nat Commun. 2021 May 21;12:3020. doi: 10.1038/s41467-021-23294-7 (PMC8140076; doi:10.1038/s41467-021-23294-7)
Supplement: Supplementary file 10 — Description of Additional Supplementary Files [file 41467_2021_23294_MOESM10_ESM.pdf]

Title: Supplementary Movie 1
Description: Z-stack of day 13 EMLO SOX2/GATA6, 40x magnification, 7 frames/s. See also Fig. 2d.

Title: Supplementary Movie 2
Description: Z-stack of day 20 EMLO TUJ1/FOXA2, 40x magnification, 7 frames/s. See also Fig. 2g.

Title: Supplementary Movie 3
Description: 3D rotation of EMLO TUJ1 immunostain in ME compartment from Fig. 5d using Imaris software.

Title: Supplementary Movie 4
Description: Fluo-4 AM in EMLO-derived adherent neuronal cultures (BrainPhys baseline). 20x magnification, 17 frames/s, 50 ms exposure at 200 ms interval acquisition (1.5 min).

Title: Supplementary Movie 5
Description: Fluo-4 AM in EMLO-derived adherent neuronal cultures (1 µM DAMGO). 20x magnification, 17 frames/s, 50 ms exposure at 200 ms interval acquisition (1.5 min).

Title: Supplementary Data 1.
Description: Key resources. This supplementary data file provides key resources including detailed primary antibody information, chemicals, commercial kits, experimental models and cell lines, software, and other equipment.

Title: Supplementary Data 2.
Single cell sequencing annotated gene lists. This supplementary data file provides comprehensive gene lists used to annotate the day 16 H3.3.1 EMLO single cell sequencing data set.
